# Evaluation of the implementation process of POC-CRP devices in managing adults with cough in general practice: insights from a focus group study

**DOI:** 10.1186/s13690-026-01868-5

**Published:** 2026-03-03

**Authors:** Annelien Dankaerts, Stefanie Goeman, Marina Digregorio, Frauke Willemsens, Lauren Vermetten, Lieve Van Hoovels, Jan Y Verbakel

**Affiliations:** 1 Leuven Unit for Health Technology Assessment Research (LUHTAR), Department of Public Health and Primary Care, KU Leuven, Leuven, Belgium; 2 Academic Centre for General Practice, Department of Public Health and Primary Care, KU Leuven, Leuven, Belgium; 3 Faculty of Medicine, KU Leuven, Leuven, Belgium; 4 Research Unit of Primary Care and Health, Department of General Medicine, Faculty of Medicine, University of Liège, Liège, Belgium; 5 Department of Microbiology, Immunology and Transplantation, KU Leuven, Leuven, Belgium; 6 Department of Laboratory Medicine, AZORG Hospital, Aalst, Belgium

**Keywords:** Point-of-care testing (POCT), C-reactive protein (CRP), Ambulatory care, Primary care, General practice, General practitioners, Antibiotic stewardship, Antibiotics, Infections, Acute cough

## Abstract

**Background:**

Although acute respiratory tract infections are typically self-limiting, general practitioners (GPs) frequently prescribe antibiotics. C-reactive protein (CRP) point-of-care testing (POCT) has been shown to significantly reduce antibiotic prescribing and is cost-effective for adults with acute cough. However, before nationwide implementation in Belgium, organizational barriers must be addressed.

**Aim:**

to explore GPs’ experiences in a pilot project, using POC-CRP devices in general practice for adults with acute cough.

**Methods:**

Following a national CRP POCT pilot study, four multidisciplinary, semi-structured focus groups were conducted with 32 Belgian GPs and relevant stakeholders, whose role was limited to offering technical clarification when needed. Transcripts were analyzed using reflexive thematic analysis.

**Results:**

Across four focus groups, we identified four overarching themes.

i. Reduced diagnostic uncertainty: CRP POCT is used as an objective tool to guide clinical decisions in cases of diagnostic uncertainty and to manage patient expectations. Doctors highlighted its usefulness across various conditions, especially in children.

ii. Seamless integration with reliable support: The test was well integrated into routines, with GPs multitasking during the short waiting time. The device’s speed, simplicity, and small blood sample volume were crucial. GPs stressed the need for robust quality control and manufacturer support.

iii. Facilitated decision making and patient communication: CRP POCT, used in addition to clinical judgment, supported clinical decisions, seemed to reduce unnecessary antibiotics, and reassured both doctors and patients. It also encouraged peer consultations when results were unclear.

iv. Financial and reimbursement concerns: High test costs and lack of reimbursement remain key barriers to wider adoption.

**Conclusions:**

Findings suggest that the tailored implementation of CRP POCT in Belgium can be effective if key aspects such as ease of use, seamless workflow integration, and decision-making support are prioritized. This approach aims to reduce inappropriate antibiotic prescribing and support antimicrobial stewardship efforts.

**Supplementary Information:**

The online version contains supplementary material available at 10.1186/s13690-026-01868-5.

**Table:** 

Text box 1. Contributions to the literature
• This study provides new insights into the real-world use of C-reactive protein point-of-care tests (CRP POCT) in Belgian general practice, highlighting both opportunities and challenges for wider implementation.
• It shows CRP POCT can fit efficiently into GP workflows, reduce diagnostic uncertainty, support communication with patients, and promote responsible antibiotic use, contributing to antimicrobial stewardship.
• The findings identify key conditions for successful adoption, such as clear guidelines, reimbursement mechanisms, and strong support from both manufacturers and laboratories, offering guidance for policymakers and health systems considering nationwide rollout.

## Background

Antimicrobial resistance (AMR) is listed by the WHO as one of the top 10 global public health and development threats [1]. AMR occurs when pathogens no longer respond to antimicrobial drugs, resulting in an increased risk of treatment failure and recurrent infections [2]. As a result, it plays a significant role in the rising rates of morbidity and death and, ultimately, rising healthcare costs [3]. An estimated 4.95 million deaths have been associated with bacterial AMR in 2019, including 1.27 million deaths directly attributable to bacterial AMR [4]. By 2050, AMR is estimated to be accountable for over 10 million deaths per year, with associated costs as high as 100 trillion dollars if no action is taken [4–6].

One of the main causes of AMR is the use of antibiotics in human medicine [7, 8]. It is estimated that over 90% of all antibiotics used in humans are prescribed in primary care, most often by general practitioners (GPs) [9]. Lower respiratory tract infections (LRTIs) are among the most common acute illnesses for which patients consult their GP and for which antibiotics are prescribed in primary care, even though these infections are usually self-limiting and do not require antibiotics [9, 10].

LRTIs comprise infections of the lower airways (acute bronchitis) and lung(s) (pneumonia), with the majority of cases consisting of acute bronchitis. They often include a combination of symptoms such as sore throat, cough, fever and shortness of breath [11]. Although most LRTIs are viral and antibiotics are therefore not recommended for patients with acute bronchitis according to the Belgian Antibiotic Policy Coordination Committee (BAPCOC) guidelines, data suggest that antibiotics are prescribed in 51% to 80% of bronchitis cases [12, 13].

A key factor contributing to inappropriate antibiotic prescribing by GPs for RTIs or patients with acute cough is the challenge of distinguishing self-limiting from potentially deteriorating cases, based solely on patient history and clinical examination [11]. The low predictive value of clinical assessments and the inherent diagnostic uncertainty often lead to overprescription of antibiotics [14].

Despite extensive efforts to reduce inappropriate antibiotic prescribing in Belgian primary care, the targets outlined in the National Action Plan on AMR, have not yet been met [15]. Reducing unnecessary antibiotic prescribing is essential to combat the growing threat of AMR. Diagnostics like point-of-care (POC) C-reactive protein (CRP) testing can assist GPs’ clinical decision-making during patient consultations. POC-CRP tests are designed for in vitro diagnostics, measuring CRP levels in human capillary (finger prick) whole blood, venous whole blood or plasma, using a POCT analyzer. CRP, an acute-phase protein produced by the liver in response to inflammation or injury, is a rapid and reliable marker that provides insight into the severity of an infection [16]. It reflects the extent of inflammation or tissue damage [17], aiding clinicians in determining whether an infection is self-limiting and assessing whether antibiotics would benefit the patient [17]. CRP can also help differentiate between bacterial and viral infections, although its diagnostic accuracy for identifying bacterial infections remains limited, and assist in monitoring the effectiveness of antibiotic therapy [18–21].

Clinical studies in primary care have demonstrated that using CRP POCT to guide antibiotic prescribing significantly reduces inappropriate prescriptions without compromising patient safety [16, 20, 22–24]. In adults with acute cough, this intervention has proven to be both cost-effective and effective over the long term [22, 24–26]. In several European countries, including the Netherlands, POC-CRP testing is included in guidelines for managing acute cough and respiratory infections in general practice [27–30].

In Belgium, however, these tests have not yet been widely implemented or rigorously evaluated. Research indicates that successful national adoption of CRP POCT requires tailored organizational and implementation strategies [17]. Sustainable implementation to combat AMR also necessitates the engagement of healthcare stakeholders [17]. Qualitative research plays a key role in understanding how to integrate evidence-based practices effectively, but few studies have explored the clinical indications, rationale, effects, training, usability, and broader integration and implementation of CRP POCT in Belgian general practices [31].

This study aims to address this gap by conducting focus group discussions with key stakeholders to assess the feasibility and identify barriers and facilitators related to implementing of POC-CRP devices in Belgian general practices. The findings will inform strategies for integrating POC-CRP devices into primary care while evaluating the organizational changes needed for potential nationwide implementation.

## Methods

### Study design and setting

This research employed a qualitative approach, using semi-structured focus groups with key stakeholders for the implementation of CRP POCT in primary care. Stakeholders in this study included GPs, GP trainees, representatives from companies distributing POC-CRP devices, and clinical biologists. These stakeholders participated in a pilot project involving clinically validated POC-CRP devices (QuikRead go easy CRP test (Aidian Diagnostics, Espoo, Finland), Cobas b101 CRP test (Roche Diagnostics, Mannheim, Germany) and Afinion 2 CRP test (Abbott, Oslo, Norway). These devices were installed in 24 GP practices, 1 nursing home and 1 GP out-of-hours center across Belgium, with the necessary IT support provided. The participating sites were recruited by one of the four central laboratories they were linked to: Department of Laboratory Medicine AZORG (Aalst), Medical Laboratory Medina (Aalter), LHUB-ULB (Brussels) and Laboratoires Cliniques St-Luc (Brussels) by contacting affiliated practices with study details and inviting those interested to participate. The devices and the laboratory information systems were connected *via* middleware (Roche Cobas Infinity POC) in a subset of practices (15 practices connected to AZORG lab and 1 to 3 practices for the other central labs). The clinical laboratory validated the results, which were then integrated into the GP’s electronic medical record (EMR) through the established channels. The pilot project began with a plenary training session for end-users, delivered by the study team, manufacturers and clinical laboratories. This session covered the clinical application of POC-CRP testing, study protocols, device usage and included a hands-on component. After installation, GPs and their staff conducted all study-related activities including recruiting eligible patients with acute cough, obtaining the informed consent form, performing the POC-CRP test using finger prick blood samples, explaining the results to the patient, and conducting routine quality controls (QC) checks. Each GP used one to three POC-CRP devices over approximately three months, from September to December 2023.

Four focus groups were held between January and March 2024 to explore GPs’ experiences with the CRP devices, following the methodology by Krueger et al. [32]. The reporting of this study adheres to the Consolidated Criteria for Reporting Qualitative Research (COREQ) checklist (Additional file 1 (I)), and the protocol is provided in Additional file 1 (II).

### Recruitment and selection of participants

We contacted participants of the CRP-POCT pilot project via email to join a focus group at a clinical laboratory in Aalst, Aalter or Brussels. Due to high interest in Aalst, two focus groups were organized, one of which was conducted online due to unforeseen weather conditions. Representatives from companies distributing POC-CRP devices selected one or more focus groups to attend. The company representatives were present solely to provide technical clarification when needed, such as answering factual questions about device functioning, error messages or quality control procedures, and they did not participate in the substantive discussion. Laboratory professionals attended for the same reason, providing information related to system linkage and quality control, and they also did not engage in the general discussion. Participant demographics were collected *via* email post-hoc.

### Focus group discussions

Focus groups included three Dutch-speaking groups (Aalter, Aalst), moderated by SG, with AD as an observer, and one French-speaking group (Brussels), moderated by MD with SG as an observer. Field notes were taken by the observer to identify key elements, inform analysis, and refine subsequent focus groups.

The discussion followed a semi-structured interview guide (Additional file 1 (III)) to ensure that all critical areas were addressed, while still allowing participants to add additional discussion topics. The topic guide was initially developed in Dutch based on previous research. Several authors of this paper, with diverse professional expertise, worked on refining the final version of the topic guide, which was subsequently translated and reviewed with French-speaking researchers. The following topics were covered: (1) overview of the purpose and rationale of the focus group and signing of the informed consent form (2), participant introductions (3), prior experiences with POC-CRP devices, including facilitators and barriers (4), installation, organization and feasibility of the devices (5), handling technical issues and point of contact (6), usage patterns and rationale for employing the device. The interview guide was created in Dutch and then translated and discussed with the French speaking researcher.

### Data analysis

All focus groups were audio-recorded and transcribed verbatim using Amberscript (details not available). The transcripts were subsequently reviewed by a researcher who was a native speaker of the FG language, pseudonymised, and translated into English using Deepl GmbH Pro (Berlin, Germany), as the analysis was conducted in English.

A reflexive thematic analysis was performed guided by a realist and constructionist framework following the approach by Braun and Clarke [33, 34]. The primary aim of this analysis was to develop an explanatory framework to understand GPs’ experiences with implementing POC-CRP devices in primary care. First, team members (AD, MD, SG, FW and LV) familiarized themselves with the data. Each focus group was then analyzed individually, followed by team meetings to discuss findings after each session. During this process, the research team remained aware of how their personal experiences might influence interpretations and worked collaboratively to delimit how they interpreted the data. Remarks or discrepancies were discussed in the in-between meetings to help them separate the participants’ experiences from her own and to ensure the integrity of the analysis.

Focus groups conducted in Dutch were primarily coded by Dutch-speaking researchers, while the French-language focus group was led by a French-speaking principal coder. This ensured that the lead coder always shared the native language of the participants. Additional coders and reviewers, regardless of their native language, participated in all groups to enhance reflexivity and ensure consistency. The role of each coder, except for AD, varied across focus groups, contributing to a comprehensive and balanced analytical process. AD coordinated and revised the coding during team meetings before analysis proceeded to the next focus group. From the second focus group onwards, the research team involved in analyzing the results constructed themes inductively through collaborative discussions. This process was supported by a codebook containing definitions and examples. The resulting codebook was then used to code the subsequent focus groups. Both the codebook and themes were iteratively reviewed and refined. All analyses were performed using QSR NVIVO software version 14 (QSR International Pty Ltd, Melbourne, Australia).

Following the analysis, participants were invited to validate the findings through a member-checking process [35, 36]. Although member checking does not provide definitive validation, it can offer a useful indication of whether the themes aligned with participants’ perspectives [37]. They received the final list of themes via email and were asked to rate their agreement on a 6-point Likert scale (from strongly disagree to strongly agree). Participants could also provide additional comments in a free text format. Feedback from 21 participants indicated strong agreement with the main focus group themes (Additional file 1 (IV)).

During manuscript writing, Dutch and French quotes were translated to English using DeepL software. To ensure that the translations accurately reflected participants’ intended meanings, the English quotes were backtranslated into Dutch or French and reviewed by the research team.

### Reflexivity statement

AD (MSc.) and MD (MSc., PhD) are female researchers with a background in biomedical sciences. AD is currently a PhD researcher, while MD is working as a postdoctoral researcher. As researchers with a biomedical sciences background rather than general practice, AD and MD do not have direct experience with electronic medical records (EMRs) or POC-CRP devices. Consequently, their perspectives are shaped primarily by their research expertise, which may differ from the practical experiences of GPs. SG, FW and LV are female GPs in training. JYV (Prof., MD) is a male GP with 16 years of clinical experience. LVH (PharmD, PhD) is a female clinical biologist. All team members were born in Belgium. Prior to the start of the study, AD, MD, FW and JYV already had experience with qualitative research methods. SG, LV and LVH did not have any experience in qualitative research yet but attended other focus groups to observe, followed pertinent lectures and reviewed relevant literature. There was no relevant relationship between the researchers and the participants prior to the commencement of the study.

### Number of participants

Out of 108 contacted stakeholders involved in or affiliated with the CRP POCT pilot project (GPs, GP trainees, laboratory professionals and manufacturer representatives), 58 expressed interest in participating in the focus group discussions, 37 confirmed, and 32 ultimately participated. Reasons for non-participation included: scheduling conflicts (*n* = 3), personal reasons (*n* = 1) or prior focus group participation (*n* = 1). Participants included 19 GPs, 1 GP in training, 5 manufacturer’s representatives, and 7 laboratory professionals (Table 1). Each focus group included a convenience sample of 6–12 participants, lasting 29–71 min (average: 53 min). Thematic analysis produced four intersecting themes (Table 2). Details of the coding tree and quotes in the original language are provided in Additional file 1 (V, VI).

**Table 1:** Characteristics of stakeholders participating in focus groups on CRP POCT (2024) in Belgium

	N (numbers)	
Gender		
Male	10/32	
Female	22/32	
Age (years old)		
20–30	5/32	
31–40	6/32	
41–50	13/32	
51–60	4/32	
60+	4/32	
Clinical experience (years)		
0–5	5/32	
6–10	4/32	
11–20	6/32	
21–30	2/32	
30+	3/32	
Not applicable	12/32	
Specialty		
General Practitioner	19/32	
GP trainee	1/32	
Representatives of the lab	7/32	
Representatives of the CRP POCT manufacturer	5/32	
Practice setting		
Group practice (2 or more doctors)	20/32	
Solo practice	0/32	
Not applicable	12/32	
Degree of urbanisation^a^		
Rural	0/32	
Town-suburb	16/32	
City	4/32	
Combination	0/32	
Not applicable	12/32	
Number of participants per FG (date and location)^b^		
FG1 (17 January 2024 – Aalst, Department of laboratory Medicine AZORG)	9/36	
FG2 (5 March 2024 – Brussels, Medibois Medical Center)	12/36	
FG3 (11 March 2024 – Aalter, Medical Laboratory Medina)	10/36	
FG4 (27 March 2024 – Department of laboratory Medicine AZORG)	6/36	
Native language		
Dutch	22/32	
French	10/32	

*FG* focus group interview, *GP* general practitioner

^a^The Degree of Urbanisation (DEGURBA) is a classification that indicates the character of an area. It is based on the share of local population living in urban clusters and in urban centres, and is classified into three types of area: “city”, “town/suburb”, “rural”

^b^Four manufacturer’s representatives attended two focus groups. This results in a total of 36 participations across 32 unique individuals

## Results

**Table 2:** Thematic framework derived from focus groups on the implementation of CRP POCT (2024) in Belgium

Themes	Subthemes
Theme 1: CRP POCT is an objective, non-invasive tool that reduces diagnostic uncertainty and manages patient expectations	1.1 CRP POCT as an objective tool to facilitate therapeutic decision making
	1.2 CRP POCT is used in cases of diagnostic uncertainty and to manage patient expectations
	1.3 CRP POCT should be used over and above clinical examination
	1.4 CPR POCT can be used for various clinical indications and in diverse contexts
	1.5 CRP POCT instead of general blood test in children
Theme 2: Easy-to-use POCT with reliable support integrates well in existing workflow	2.1 Installation, adaptation, cooperation, frequency of use and quality control
	2.2 Integration of the CRP POCT in the GP’s workflow
	2.3 Most important factors for optimal integration in workflow
Theme 3: CRP POCT supports decisions and strengthens patient communication, fostering antibiotic stewardship	3.1 Interpretation of the (surprising) outcome of the CRP test, leading to co-consultation
	3.2 Consequences of the test for GPs and patients
Theme 4: GPs expressed concerns regarding cost and reimbursement of CRP POCT	

### Theme 1: CRP POCT is an objective, non-invasive tool that reduces diagnostic uncertainty and manages patient expectations

#### CRP POCT as an objective tool to facilitate therapeutic decision making

Physicians often referred to CRP POCT as an objective tool that can serve as a valuable complement to gut feeling and clinical examination. They highlighted the synergy between these approaches, emphasizing how the POC-CRP result aids GPs in distinguishing between viral and bacterial infections. This test also facilitated therapeutic decision-making, particularly in cases where patients express their symptoms in diverse ways or have different perceptions of illness.

*F2P4. “And at clinical level… I think it provides us with something more objective. In general practice*,* it’s true that we’re very good at relying on gut feeling and clinical examination and also sensing how people feel. But the CRP allows for a certain objectification of things.”*

#### CRP POCT is used in cases of diagnostic uncertainty and to manage patient expectations

Participants reported using the POC-CRP test particularly in situations of diagnostic uncertainty and when an additional argument was needed to convince patients that antibiotics were unnecessary. Physicians noted that they commonly used the test when they encountered challenges in their diagnostic process, had doubts, or sought to narrow down the differential diagnostics, referred to as operating in “the grey zone.” The test was particularly beneficial in cases where there was uncertainty between viral and bacterial infections, especially when deciding whether to prescribe antibiotics. Additionally, physicians used the test when they had a gut feeling that something might be wrong or when they sought confirmation or reassurance. When clinical examination findings and CRP test results aligned, it offered physicians a greater sense of reassurance (Table 3).

Another reason for performing the test was to reassure or convince patients that antibiotics were not needed. When patients insist on antibiotics, the CRP value provided a clearer explanation compared to simply stating, “it’s a viral infection.” Moreover, patients felt that the doctor had acted and had no doubts. However, doctors emphasized that this should not be the sole justification for conducting the test.

*F4P1. “[…] I used it mainly in two ways. On the one hand*,* it [the CRP-test] served as a tool to sometimes convince patients not to take antibiotics*,* especially when they requested them*,* to be able to throw a kind of extra argument. On the other hand*,* in borderline cases where I was clinically concerned about a possible serious infection*,* it helped either confirm or rule out my clinical impression.”*

#### CRP POCT should be used over and above clinical examination

According to GPs, it remains important not to solely rely on the CRP value but to ensure the patient is thoroughly assessed. The POC-CRP value should always be interpreted in conjunction with clinical examination findings. If a patient appears seriously ill but has a low POC-CRP value, it is important to provide follow-up advice and encourage the patient to return if symptoms worsen, considering an inherent delay in CRP level increase. In any case, clinical examination remains the cornerstone of the GPs’ consultation, with CRP POCT serving as a supportive tool (Table 3). If the clinical examination is clear, there is no need to use the CRP device, according to the physicians.

#### CRP POCT can be used for various clinical indications and in diverse contexts

While many doctors frequently expressed their enthusiasm and confirmed the added value of CRP POCT in routine care, they also identified situations where they deliberately chose not to perform the CRP test. For instance, when the diagnosis was clear to both the doctor and patient, in cases of early fever or cough, when pressed for time, or when more comprehensive information was required, leading to a general blood test instead.

GPs explained that within the study context, they most often used the test for adults with a prolonged cough or an unusual-sounding cough with limited clinical findings. The test proved especially useful during November and December for such cases. However, they emphasized that the test was not used for all patients with a cough. Only one doctor reported using the test for every patient with acute cough due to the study’s scope but acknowledged that this often felt unnecessary.

Notably, doctors mentioned using the test for indications beyond the study’s scope, such as erysipelas, urinary tract infections, pneumonia, diverticulitis and appendicitis. They also used the CRP test in contexts such as nursing homes (though less frequently than expected), during home visits where a rapid diagnosis was required, and in children.

*F4P2. “People appreciated that a whole blood sample wasn’t needed. Even those who were afraid of needles accepted it. So you really have something [valuable] there.”*

#### CRP POCT instead of general blood test in children

The use of the test in children was a recurring topic in every focus group. According to GPs, the test was especially valuable for children because of the increased diagnostic uncertainty and the need to quickly determine whether to refer the child to a pediatrician. Even in cases where the doctor did not suspect anything serious, it could serve to reassure parents. A POC-CRP test is easier to perform in children, as it is less painful and quicker than a general blood test. Moreover, the results are available immediately, enabling prompt decision-making without the need for a follow-up contact.

*F1P8. “We focused on adults with coughs*,* but I think the added value in children is huge. You don’t have to do heavy blood sampling*,* which they [children] don’t like. A simple finger prick is more acceptable to them.”*

### Theme 2: Easy-to-use CRP POCT with reliable support integrates well in existing workflow

#### Installation, adaptation, cooperation, frequency of use and quality control

Several doctors initially required some time to become accustomed to the device, but once familiar, they generally found it easy to use. Interestingly, general practice trainees adapted to the device more quickly and integrated it into their workflow more effectively. They also reported using it more frequently and stated they would prefer to continue using the device. In contrast, more experienced doctors were sometimes slower to adapt to the device, since they are accustomed to working without it. Another reason mentioned was that younger GPs required more reassurance regarding their clinical assessment of the patient, whereas older GPs relied more on their own expertise.

*F3P8. “I also noticed that*,* in my practice*,* it was mainly the younger doctors who used it a lot*,* although I used it the most myself*,* I think (everyone laughs). This was mostly due to diagnostic uncertainty. The slightly older doctors tended to use it a little less and relied more on their clinical experience.”*

The frequency of device use varied widely among doctors, ranging from infrequent use to daily use, with some reporting usage as high as 7–8 times a day. One participant questioned whether the high number of tests was driven by availability rather than its genuine necessity. However, many doctors indicated that they would miss the device if it were no longer available. Based on laboratory data retrieved after the focus groups, the devices were used on average 150 times per practice over the three-month period.

The installation and implementation of the devices were described by nearly all doctors as very smooth. Any issues that arose were promptly resolved by the companies and labs, which were easily accessible by phone and email. This swift support was highly appreciated by the GPs (Table 3). The most reported problems included IT and network issues, connectivity errors between the lab and the device, error messages, and temperature-related issues (e.g. the device being too warm or cold) (Table 3). However, some participants emphasized that they encountered no technical issues at all. Additionally, the labs provided QC supervision and supplied extra materials if needed.

*F2P12. “We had several points of contact […] whenever I called for help*,* we received immediate support. […] It’s clear that if these machines are going to be implemented*,* having such invaluable people around is essential.”*

According to a lab representative, an insufficient number of QC tests were performed, even within the current study framework. A possible solution proposed was to program the device to block usage after a certain number of tests, requiring a QC test before becoming operational again. GPs admitted that QCs tests were not always performed, either because they were considered too much hassle or simply forgotten. Some doctors noted that, while it took time to get used to performing QC tests initially, they found it easy to carry out once familiar. Other general practitioners delegated the responsibility of QC tests to their practice assistants (Table 3).

*F2P9. “In general practice*,* you always have to consider whether the results are reliable*,* especially because calibration isn’t done systematically. […] We don’t have the staff or the level of rigor that laboratories have. […] Calibrating the machine isn’t complicated*,* you just need to be disciplined. It’s quick and really useful in daily practice*,* so it’s worth taking the time.”*

Experiences with the integration between laboratories and the EMR, as well as with encoding CRP results into the EMR, varied among users. Some doctors preferred manually entering the results, while others favored an automatic encoding system directly within the EMR for convenience and efficiency.

#### Integration of CRP POCT in the GP’s workflow

All doctors agreed that the test is most effectively conducted during the consultation, following the clinical examination, but prior to discussing the diagnosis and treatment plan.

The finger prick blood sampling was carried out by the GP or nurse. Some GPs had also occasionally performed a rapid test using a sample from general blood collection. The results were either reviewed by the doctor directly or relayed by the nurse or secretary. The waiting time of the test (2 to 3 min) was generally not perceived as disruptive to the consultation. Instead, it was often used to explain the diagnosis to the patient, prescribe other medications, write work certificates, perform additional tests, engage in small talk, or take a brief moment of rest. However, some GPs felt that the waiting time could be shorter (Table 3). Experience played a crucial role in seamlessly integrating the test into consultations. With frequent use, doctors became adept at timing the test and making efficient use of the waiting period.

By multitasking, several GPs felt that the test did not add extra time to the consultation but was instead seamlessly integrated into the process. Other GPs observed that, despite the test’s quick turnaround, it could still disrupt the consultation. This disruption was attributed to factors such as the timing of the test, the location of the device, or physicians checking the device before the results were available.

*F2P12. “Basically*,* it takes five minutes at most. But it’s time that can be integrated into the consultation. […] The sampling is done during the examination*,* during the test part we do something else and afterwards we look for the result. So it’s not five extra minutes. Sometimes it even saves time*,* especially when you need to explain why antibiotics weren’t prescribed. Other times it requires more explanation*,* but that’s part of the job.”*

#### Most important factors for optimal integration in workflow

GPs emphasized that the location of the POC-CRP device is critical for its ease of use. Having the device in a shared accessible space, where other GPs are not disturbed during testing, encourages its use. Also, the environment must maintain an appropriate temperature (neither too hot, nor too cold) to ensure the device operates correctly. Some GPs reported issues when the device was placed in unsuitable locations, such as a cold kitchen or under a heater. Additionally, they emphasized the importance of making the test as user-friendly, quick, and minimally invasive as possible (Table 3). Some GPs also noted that it would be helpful if samples could be preserved longer (up to 2 h), which would facilitate processing of samples collected during home visits.

*F2P1. “I think it’s the ease of use. We’re not lab assistants*,* so it really has to be easy. As I mentioned earlier*,* I think it’s important that it’s both quick and simple.”*

### Theme 3: CRP POCT supports decisions and strengthens patient communication, fostering antibiotic stewardship

#### Interpretation of the (surprising) outcome of the CRP result, leading to co-consultation

GPs reported that the test results sometimes surprised them in two main ways. Sometimes a patient who appeared seriously ill had a very low POC-CRP result; other times, a patient who seemed to have nothing serious had an unexpectedly high POC-CRP result. Otherwise, they might have probably seen the latter patient again in a few days, but now they could provide immediate care. Other times, the test often aligned with their gut feeling or intuition, offering reassurance and confidence in their clinical judgement.

*F1P8. “I was often surprised in both directions. Sometimes I thought*,* ‘This probably isn’t serious*,* but let’s check just to be sure*,*’ and then the CRP was sky-high. Other times*,* someone seemed seriously ill*,* and the result turned out to be much less alarming than expected.”*

GPs emphasized that one must be cautious of false reassurance. You should not let the POC-CRP value fully guide your decision. It remains important to observe the patient and trust your intuition, they mentioned.

*F1P1. “We shouldn’t let it fully guide us*,* but it does provide something*,* perhaps reassurance*,* that you’re thinking in the right direction.”*

Discussions or occasional confusion emerged regarding the cut-off values of the POC-CRP test. Some GPs mentioned the NICE guidelines with cut-off values of 20 and 100 mg/L, while other GPs used different thresholds (Table 3). Despite these variations, GPs agreed that the test is most useful when the result is either very low or very high. In these cases, the follow-up actions are clear. The GPs noted that the challenge occurs when the result falls within the grey zone, which they typically described as between 30 and 60 mg/L. In such cases, many GPs sought advice from colleagues. According to the participants, this dynamic encouraged co-consultation and discussion among different doctors. Some also referred to the CRP device as a “meeting point” for collaboration among GPs.

*F2P12. “What I found interesting was that it triggered a lot of informal discussion and co-consulting. For example*,* my colleague had a test result of 27 and still felt the patient wasn’t doing well*,* so she asked*,* ‘Can you come and listen? What’s your opinion?’ I found that very interesting.”*

#### Consequences of the test for GPs and patients

Besides confirming or challenging the GPs’ intuition, the test influenced whether to prescribe antibiotics. Some GPs mentioned that CRP POCT allowed them to confidently prescribe fewer antibiotics, despite initially expecting to encounter more high CRP levels, which they thought would result in increased prescribing. One doctor mentioned that, in the past, they might have provided a delayed antibiotic prescription for the weekend, but now they felt confident enough to forgo prescribing antibiotics entirely if the CRP was low. Explaining to the patient in advance that antibiotics are not needed when the CRP is low made it easier for patients to accept the decision. GPs also reported that the CRP test was well-received by patients, who appreciated the additional testing and were pleasantly surprised to receive the results immediately, rather than having to wait until the next day (Table 3).

*F1P8. “If we want to meet our targets for reducing antibiotic prescriptions*,* this is a helpful tool. It allows you to say in a more reassuring way*,* both for the patient and for yourself*,* ‘We’re not going to do it [prescribe antibiotics]. And also vice versa.”*

*F1P1. “I think it’s definitely an added value when deciding about antibiotics*,* also for your patient. If you clearly explain in advance*,* ‘If it [CRP value] is low*,* it’s viral; if it’s high*,* it may be bacterial’*,* and you come back and say*,* ‘Look*,* it’s low’*,* they [patients] have more confidence in it [the decision]. It’s more persuasive than simply saying*,* ‘No*,* you don’t need antibiotics.’ So I think that’s a real benefit for them as well.”*

### Theme 4: GPs expressed concerns regarding cost and reimbursement of CRP POCT

Although not included in the interview guide, the topic of financing and reimbursement was spontaneously raised by GPs across multiple focus groups.

One doctor mentioned they would consider investing in the device itself but noted that the cost of the individual test cassettes should not be borne by the doctor. They suggested the possibility of passing this cost on to the patient, though this was seen as suboptimal given that measuring CRP via a general blood test is nearly free. GPs generally believed that parents would be willing to pay for the test for their children, but adults might prefer to wait for general blood test results rather than incur the cost. Another doctor suggested that patients would have no issue paying a small fee for the test if its purpose and benefits were clearly explained. Suggested acceptable costs for patients ranged from €5 to €15.

Doctors using CRP devices outside the study highlighted the variation in current reimbursement practices, describing it as a “Wild West” scenario and calling for a standardized framework. Some emphasized that reimbursement would be particularly beneficial for pediatric cases, where timely results are critical for urgent decision-making (Table 3). In contrast, adults could typically afford to wait a few days for general blood test results. There were mixed perspectives on the broader economic impact: one doctor argued that reimbursement for the test would ultimately save healthcare costs by reducing unnecessary care, while another warned against creating financial incentives that might encourage unnecessary testing. Additionally, it was noted that offering the test free of charge could lead to overuse by patients.

Importantly, several doctors agreed that if a solution was found to cover the cost, whether through direct payment by the patient, reimbursement by the Belgian National Institute for Health and Disability Insurance (RIZIV/INAMI), or support from mixed insurance companies, this would overcome the primary obstacle to broader implementation (Table 3).

*F1P8. “I would find it a certain added value to have that*,* a device in practice. […] the question of course is: how are you going to finance that? Okay you can say I’m going to charge each patient the cost of a test. That’s possible but that might not be optimal*,* because if you only prick CRP via the lab*,* then it’s basically free. So if a solution is found for that*,* I think that*,* of course*,* the way is wide open to roll that out everywhere.”*

*F3P2. “I think the purchase of the device could be seen as an investment*,* when you think*,* ‘Okay yes*,* I think this is worth implementing’. I don’t necessarily think that should be passed on to the patient. But if each cassette is then each time 5 euro*,* yes*,* then you immediately have a serious investment. And that makes me think*,* ‘Is it actually our responsibility to pay this?’”*.

**Table 3:** Facilitators and barriers for broader implementation of CRP POCT mentioned by GPs during focus groups (2024) in Belgium

Theme	Facilitators to adoption of CRP POCT in primary care	Barriers to adoption of CRP POCT in primary care
Theme 1: Reduced diagnostic uncertainty	Added value for GP’s: reassurance	Avoid CRP dependance
		Cannot be used as a standalone test
Theme 2: Seamless integration with reliable support	Device satisfaction	No shared location of the CRP device
	Minimally invasive method (especially helpful in children)	Quality control
	Ease of use and speed	Technical problems
	Support from the lab and companies	Disruption or prolongation of the consultation
Theme 3: Facilitated decision making and patient communication	Added value for patients: feeling something is done	Lack of clear guidelines
Theme 4: Cost and reimbursement concerns	Easier accessible than CRP test with pediatrician	High prices of test cartridges and no reimbursement

## Discussion

### Main findings

This focus group study evaluated the implementation of POC-CRP devices in Belgian general practices for managing adults with acute cough. CRP POCT supports diagnostic decisions, complementing gut feeling and clinical examination. Doctors highlighted its usefulness across various clinical indications and contexts, particularly in children, as it offers a less invasive method to assess infection severity. The test was generally well integrated into GP workflows, with doctors finding ways to multitask during the short waiting time. While CRP POCT helped confirm or reassess diagnoses, doctors emphasized that it should enhance rather than replace clinical judgment. The test also encouraged collaboration, prompting peer consultations when results were inconclusive. Doctors indicated that CRP POCT may have contributed to a reduction in unnecessary antibiotic prescribing by reinforcing their confidence in the decision to withhold antibiotics. Additionally, it provides GPs with a tool to support their communication with patients, helping them justify the decision to withhold antibiotics. Challenges for large-scale implementation include clear guidelines on cut-off values and indications for using CRP POCT, quality control compliance, and continued technical support from labs and companies, as well as the development of a coherent reimbursement model to ensure accessibility without overuse.

### Strengths and limitations

This study provides in-depth information on the implementation of POC-CRP devices in general practice, drawing on GPs’ experiences while being embedded in a broader collaborative pilot context. The involvement of laboratory professionals and industry representatives in device installation, system integration, and technical support throughout the pilot contributed important operational and organizational insights, strengthening the relevance of the study for future large-scale implementation. Conducting the study in a real-world general practice setting enhances the applicability of the findings to daily clinical practice. Further, the interview guide was developed by the research team, which included GPs, to ensure clinical relevance. Coding was performed by researchers with diverse backgrounds, reducing bias and strengthening validity. Additionally, reflexive thematic analysis was used to identify key themes, allowing for continuous refinement of the findings while encouraging reflection on the researchers’ assumptions. The focus groups were reported according to the COREQ checklist, ensuring transparency and completeness in qualitative research reporting. It should be pointed out, however, that our study was conducted in Flanders and Brussels, with predominantly female and urban-based participants, all practicing in group practices. As rural and solo practices were not represented, the analysis may lack insights specific to these contexts within the target population. In addition, while the device was tested in nursing homes and out-of-hours services, professionals from these settings did not take part in the focus groups. An inherent limitation of this study with voluntary participation is selection bias, which in our case was present both at the level of participating practices in the pilot study and individual physicians participating in the focus groups. The GPs included were likely already interested in CRP testing and diagnostic innovations, potentially leading to a more favorable attitude toward its implementation. Recall and social desirability bias may also have influenced responses, as participants might have an inaccurate recollection of past experiences or have the tendency to provide socially favorable answers. In addition, the presence of non-clinical stakeholders may have influenced group dynamics or introduced bias, even though their contribution to the discussion was limited. Furthermore, the study relies solely on GPs’ reports on patients’ attitudes, which may not fully capture reality, as physicians might unintentionally interpret patients’ reactions based on their own perspective. Different POC-CRP devices were used across practices, introducing potential variability in user experiences. While this may affect consistency, it also reflects real-world diversity in device availability and operational challenges. Additionally, the absence of national guidelines for CRP testing in Belgium led to uncertainty about cut-off values, causing variation in interpretation of CRP values and decision-making criteria. Lastly, reflexivity must be considered, as researchers’ backgrounds and prior knowledge may have influenced data collection and interpretation. To mitigate this, coding was performed by multiple researchers with diverse expertise, and regular discussions were held to minimize bias in interpretation.

### Comparison with existing literature

Our findings align with previous studies, who reported that CRP POCT increases diagnostic certainty and provides reassurance for both clinicians and patients but should not be used as a standalone diagnostic tool [38, 39]. CRP POCT helps manage patient expectations regarding antibiotics, facilitating shared decision-making [40, 41]. Additionally, in a cluster randomized trial, high patient satisfaction was reported, particularly when CRP POCT was combined with clear physician communication strategies [42] This aligns with our findings, where GPs reported that POC-CRP supported communication and patient acceptance of non-prescribing decisions, especially when clearly explained. Studies on CRP POCT in children show mixed results: some GPs find it useful, while others are cautious due to unclear cut-off values and vulnerability of pediatric patients [43, 44]. CRP POCT’s effect on antibiotic use in children remains inconclusive [45]. Similar to our findings, other studies have highlighted that the ‘grey zone’ of CRP values between 20 and 100 mg/L remains challenging, reinforcing the need for clear guidelines to support clinical decision-making [17, 46, 47]. In addition, training clinicians in the use and interpretation of CRP testing is essential to ensure proper test utilization and avoid over-reliance [47]. Workflow integration was smooth once GPs adapted, with waiting time used for patient education, which aligns with findings that POCT improved communication and consultation flow [38]. Ensuring seamless POCT workflow integration requires adequate infrastructure and support from laboratories and manufacturers, including technical support, device maintenance, and quality control systems, which is consistent with previous studies [48–50]. Focus group participants reported prescribing fewer antibiotics when using CRP POCT, suggesting a potential contribution to antimicrobial stewardship, as noted in earlier literature [24, 42, 51]. The experiences of other countries, such as the Netherlands and Norway, where CRP POCT is incorporated into guidelines and clinical practice, suggest that structured implementation can enhance its impact on prescribing behaviors [39, 50]. Financial barriers remain a significant challenge for CRP POCT adoption, with the absence of a reimbursement model identified as a major obstacle to widespread implementation [48, 52] Clear reimbursement policies are also crucial to prevent financial burdens on both clinicians and patients [17, 22, 30, 50].

### Implications for clinical practice and future research

The implementation of CRP POCT in general practice has the potential to enhance diagnostic confidence and patient communication, particularly in pediatrics, where it offers a less invasive alternative to general blood tests. Training programs must be optimized to ensure seamless adoption, while further technological improvements should focus on making tests faster and requiring even less blood. To facilitate broader integration of CRP POCT in primary care, efforts should focus on practical enablers such as clear national guidelines and coherent reimbursement mechanisms that support appropriate use without encouraging overuse. Support from central laboratories to ensure quality control, guarantee test accuracy, and provide a point of contact when errors occur, can help reduce the burden on GPs.

Future research should explore the broader application of CRP testing, particularly in children, where diagnostic uncertainty is high. Studies on patient outcomes and satisfaction, as well as economic evaluations, are essential to determine cost-benefit and inform reimbursement strategies. In addition, research should explore implementation in varied primary care settings such as rural, solo, and out-of-hours practices. Efforts should be made to include GPs who are less engaged with or unfamiliar with CRP testing. Finally, long-term research should assess the impact of CRP-guided decision-making on reducing antibiotic resistance, showing how it fits within broader antimicrobial stewardship efforts. Addressing these areas will help integrate CRP testing more effectively into routine practice, enhancing both diagnostic accuracy and responsible antibiotic use.

## Conclusion

This study underlines the potential of CRP POCT in supporting clinical decision-making for GPs managing adult patients with acute cough.

The findings emphasize the test’s value in reducing diagnostic uncertainty, facilitating patient communication, and promoting more judicious use of antibiotics, thereby contributing to antimicrobial stewardship.

To optimize the broader adoption of CRP POCT in Belgium, national guidelines, streamlined workflows, consistent quality control measures, and centralized support structures must be established.

Further research should evaluate clinical outcomes, cost-effectiveness, and long-term effects on antibiotic resistance, also considering implementation in pediatric care. With appropriate support, CRP POCT can play a pivotal role in transforming diagnostic and therapeutic practices in primary care, aligning with efforts to combat antimicrobial resistance, and improve patient care.

## Supplementary Information

Additional File 1. Additional Files.

## Data Availability

The datasets supporting the conclusions of this article are included within the article and the Additional File.

## Abbreviations

GP: General Practitioner
CRP: C-reactive protein
POCT: Point-of-care testing
POC-CRP: Point-of-care C-reactive protein
AMR: Antimicrobial resistance
LRTI: Lower respiratory tract infection
RTI: Respiratory tract infection
BAPCOC: Belgian Antibiotic Policy Coordination Committee
RIZIV/INAMI: Belgian National Institute for Health and Disability Insurance
EMR: Electronic Medical Record
QC: Quality control
FG(s): Focus group(s)
COREQ: Consolidated Criteria for Reporting Qualitative Research
LUHTAR: Leuven Unit for Health Technology Assessment Research
DEGURBA: Degree of Urbanisation
FOD: Belgian Federal Public Service
FWO: Research Foundation Flanders
