# Supplementary material for: Evaluation of the implementation process of POC-CRP devices in managing adults with cough in general practice: insights from a focus group study
Source: Arch Public Health. 2026 Mar 3;84:70. doi: 10.1186/s13690-026-01868-5 (PMC13063918; doi:10.1186/s13690-026-01868-5)
Supplement: Supplementary file 1 — Additional File 1. Additional Files. [file 13690_2026_1868_MOESM1_ESM.docx]

**Additional file 1**

Evaluation of the implementation process of POCT-CRP devices in managing adults with cough in general practice: Insights from a focus group study.

**Table of contents**

I. Consolidated criteria for reporting qualitative research (COREQ): 32-item checklist 2

II. Study protocol 5

III. Interview guide 37

IV. Participant checking: results............................................................................................. 38

V. Full coding tree.................................................................................................................. 39

VI. Dutch and French quotes................................................................................................. 41

# I. Consolidated criteria for reporting qualitative research (COREQ): 32-item checklist

Developed from:

Tong A, Sainsbury P, Craig J. Consolidated criteria for reporting qualitative research (COREQ): a 32-item checklist for interviews and focus groups. International Journal for Quality in Health Care. 2007. Volume 19, Number 6: pp. 349 – 357

| **No. Item** | **Guide questions/description** | **Reported on Page #** |
| --- | --- | --- |
| **Domain 1: Research team and reﬂexivity** |  |  |
| *Personal Characteristics* |  |  |
| 1. Interviewer/facilitator | Which author/s conducted the interview or focus group? | 7 |
| 2. Credentials | What were the researcher’s credentials? E.g., PhD, MD | 9 |
| 3. Occupation | What was their occupation at the time of the study? | 9 |
| 4. Gender | Was the researcher male or female? | 9 |
| 5. Experience and training | What experience or training did the researcher have? | 9 |
| *Relationship with participants* |  |  |
| 6. Relationship established | Was a relationship established prior to study commencement? | 9 |
| 7. Participant knowledge of the interviewer | What did the participants know about the researcher? E.g., personal goals, reasons for doing the research | 6-7 |
| 8. Interviewer characteristics | What characteristics were reported about the interviewer/facilitator? E.g., Bias, assumptions, reasons and interests in the research topic | 9 |
| **Domain 2: study design** |  |  |
| *Theoretical framework* |  |  |
| 9. Methodological orientation and Theory | What methodological orientation was stated to underpin the study? E.g., grounded theory, discourse analysis, ethnography, phenomenology, content analysis | 8 |
| *Participant selection* |  |  |
| 10. Sampling | How were participants selected? E.g., purposive, convenience, consecutive, snowball | 7 |
| 11. Method of approach | How were participants approached? E.g., face-to-face, telephone, mail, email | 7 |
| 12. Sample size | How many participants were in the study? | 7; 10 |
| 13. Non-participation | How many people refused to participate or dropped out? Reasons? | 10 |
| *Setting* |  |  |
| 14. Setting of data collection | Where was the data collected? E.g., home, clinic, workplace | 7 |
| 15. Presence of non-participants | Was anyone else present besides the participants and researchers? | 7 |
| 16. Description of sample | What are the important characteristics of the sample? E.g., demographic data, date | 10-11 |
| *Data collection* |  |  |
| 17. Interview guide | Were questions, prompts, guides provided by the authors? Was it pilot tested? | 7; Suppl. M. |
| 18. Repeat interviews | Were repeat interviews carried out? If yes, how many? | 7 |
| 19. Audio/visual recording | Did the research use audio or visual recording to collect the data? | 8 |
| 20. Field notes | Were ﬁeld notes made during and/or after the interview or focus group? | 7 |
| 21. Duration | What was the duration of the interviews or focus group? | 7 |
| 22. Data saturation | Was data saturation discussed? | NR |
| 23. Transcripts returned | Were transcripts returned to participants for comment and/or correction? | 8 |
| **Domain 3: analysis and ﬁndings** |  |  |
| *Data analysis* |  |  |
| 24. Number of data coders | How many data coders coded the data? | 8 |
| 25. Description of the coding tree | Did authors provide a description of the coding tree? | Suppl. M. |
| 26. Derivation of themes | Were themes identiﬁed in advance or derived from the data? | 8 |
| 27. Software | What software, if applicable, was used to manage the data? | 8 |
| 28. Participant checking | Did participants provide feedback on the ﬁndings? | 8 |
| *Reporting* |  |  |
| 29. Quotations presented | Were participant quotations presented to illustrate the themes/ﬁndings? Was each quotation identiﬁed? E.g., participant number | 14-23 |
| 30. Data and ﬁndings consistent | Was there consistency between the data presented and the ﬁndings? | 14-23 |
| 31. Clarity of major themes | Were major themes clearly presented in the ﬁndings? | 14-23 |
| 32. Clarity of minor themes | Is there a description of diverse cases or discussion of minor themes? | 14-23 |

NR: not reported; Suppl. M.: Supplementary Material

Developed from: Tong A, Sainsbury P, Craig J. Consolidated criteria for reporting qualitative

research (COREQ): a 32-item checklist for interviews and focus groups. International Journal for Quality in Health Care. 2007. Volume 19, Number 6: pp. 349 – 357

# II. Study protocol

Internal Ref.: S67992


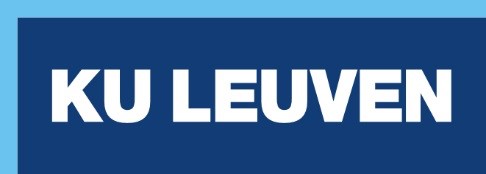


**CLINICAL INVESTIGATION PLAN (CIP)**

**Evaluation of the implementation process of POCT-CRP devices in general practice in adults with cough.**

**FOD POCT CRP project**

**Version number:** v *1.1* **– Date** *24/08/2023* **Clinical Investigation number:** S*67992* **Single Identification Number (SIN):** *Not applicable* **Sponsor**

KU Leuven

Herestraat 49, B-3000 Leuven

**Coordinating Investigator**

Prof Dr Jan Verbakel

**Confidentiality Statement**

*The information in this document is strictly confidential and is available for review to Investigators, potential*

*Investigators and appropriate Ethics Committees, Institutional Review Boards or Competent Authorities. No disclosure should take place without written authorization from the Sponsor.*

-TMP-004-1.0_28Sep2022 Page 1 of 29 Protocol template for clinical investigation Confidential & Proprietary h IMD, used within CE-label

**LIST OF PARTICIPATING SITES**

**(if applicable)**

**List Of Participating Sites Principal Investigator**

| Medicasse | Dr Vermeulen |
| --- | --- |
| Vronemeers | Dr Anouk De Raedemaeker |
| De Sadeleer BART | Dr Hannelore De Sadeleer |
| Doktersteam Welle | Dr Hannelore Van Droogenbroeck |
| De Dender | Dr Annelies De Vuyst |
| WACHTPOST AALST | Dr Patrick De Coninck |
| LATOIR ANNELIES | Dr Annelies Latoir |
| TONDELIER | Dr Celine De Bruyne |
| VIVE | Dr Marc Verstraeten |
| VAN HOECKE | Dr Peter Van Hoecke |
| ROWIES | Dr Marc Rowies |
| COVENS | Dr Corinne Covens |
| MEDISCH HUIS LENNIK | Dr Dirk Van De Sype |
| DE MUNTER | Dr Hanne Van Den Eede |
| BEECKMAN | Dr An Beeckman |
| Huisartsenpraktijk Petegem | Dr De Backer Stefanie |
| Praktijk Bouvelo | Dr Caroline Erregat |
| Groepspraktijk Varendries | Dr Bruggeman |
| Groepspraktijk Lotenhulle | Dr Huyberechts |
| Huisartsenpraktijk Flora | Karim De Boever |
| Groepspraktijk Huisartsen Latem | Dr Vermeulen |
| Cabinet Médical Dr Decroly | Dr Decroly |

**SIGNATURES**

*Form to be* ***repeated for every Participating Site*** *and signature of Prinicple Investigator. There can only be one (1) single Coordinating Investigator, while multiple Principal Investigators can be applicable (one (1) Principal Investigator per Participating Site) depending on the amount of Participating Sites.*

Title: **Evaluation of the implementation process of POCT-CRP devices in general practice in adults with cough.**

CIP: FOD POCT CRP project

The undersigned confirm that the following CIP has been acknowledged and accepted and that they agree to conduct the Investigation in compliance with the approved CIP (and any subsequent amendments if applicable) and will adhere to the principles outlined in the Declaration of Helsinki, applicable Good Clinical Practices and regulations, EU General Data Protection Regulation 2016/679 (GDPR)) and ISO 14155:2020), the appropriate local legislation(s) and all other applicable legal and regulatory requirements as amended. The most stringent requirements, guidelines or regulations must always be followed.

I agree to ensure that the confidential information contained in this document will not be used for any other purpose other than the evaluation or conduct of the Investigation without the prior written consent of the Sponsor.

I also confirm that I will make the findings of the Investigation publicly available through publication or other dissemination tools, in accordance with this CIP without any unnecessary delay and that an honest accurate and transparent account of the Investigation will be given; and that any discrepancies from the Investigation as planned in this CIP will be explained.

**Coordinating Investigator**

| ………………………… | ………………………… | ………………………… |
| --- | --- | --- |
| Name & Title | Signature | Date |

**Principal Investigator (Participating Site) (Principal Investigator is the same as Coordinating Investigator in case of a monocentric investigation)**

| ………………………… | ………………………… | ………………………… |
| --- | --- | --- |
| Name & Title | Signature | Date |

**TABLE OF CONTENTS**

[2.1 Study objectives and hypotheses 14](#_Toc51496)

[2.2 Primary Endpoints 14](#_Toc51497)

[2.3 Secondary Endpoints 14](#_Toc51498)

[2.4 Design of the Clinical Investigation 14](#_Toc51499)

[2.5 Justification for the design of the Investigation 14](#_Toc51500)

[2.6 Expected Duration of the Investigation 14](#_Toc51501)

[3.1 Inclusion criteria 14](#_Toc51502)

[3.2 Exclusion criteria 15](#_Toc51503)

[4.1 Participant consent and withdrawal of consent 15](#_Toc51504)

[4.2 Selection of Participants / Recruitment 15](#_Toc51505)

[4.3 Study specific procedures 15](#_Toc51506)

[4.4 Premature discontinuation 16](#_Toc51507)

[5.1 Sample Size Determination 17](#_Toc51508)

[5.2 Statistical Analysis 17](#_Toc51509)

[5.2.1 Efficacy Analysis 18](#_Toc51510)

[5.2.2 Other Analysis 18](#_Toc51511)

[5.3 Interim Analysis and Final Database Lock 18](#_Toc51512)

[6.1 Data Collection Tools and Source Document Identification 18](#_Toc51513)

[6.1.1 Operational aspects 18](#_Toc51514)

[6.2 Archiving 19](#_Toc51515)

[7.1 Ethics Committee (EC) review & reports 19](#_Toc51516)

[7.2 Regulatory Compliance 20](#_Toc51517)

[7.3 CIP / GCP compliance 20](#_Toc51518)

[7.4 Data protection and participant confidentiality 20](#_Toc51519)

[7.6 Amendments 21](#_Toc51520)

[7.7 Post-Study activities 21](#_Toc51521)

**LIST OF ABBREVIATIONS**

| **Abbreviation** | **Definition** |
| --- | --- |
| (e)CRF | (electronic) Case Report Form |
| APR | Annual Progress Report |
| CI | Coordinating Investigator |
| CIP | Clinical Investigation Plan |
| EC | Ethics Committee |
| FPFV | First Patient First Visit |
| GCP | Good Clinical Practice (latest version of ICH E6) |
| ICF | Informed Consent Form |
| ISF | Investigator Site File |
| LPLV | Last Patient Last Visit |
| PI | Principal Investigator (Participating Site) |
| TMF | Trial Master File |

**FUNDING AND SUPPORT**

**Funder Type of Financial or Non-Financial Support**

FOD Public Health Public tender FOD Public Health

FOD Public Health, Galileelaan 2/5, 1210 Brussels has issued a tender (Bestek nr. NAPAMR20202024/POCT). KU Leuven has submitted an offer and FOD Public Health as accepted. The study is thus funded by FOD Public Health.

Each of the Participating Sites (GP’s) will receive a payment of 100,00 euro for taking part in the study. Appendix 3 will contain the financial information of the GP.

The three device manufacturers will provide devices and test material for the duration of the study (contractually provided under a grant agreement with each manufacturer), but no financial support will be provided from these manufacturers.

**ROLES AND RESPONSIBILITIES**

The Principle Investigator (PI) is responsible for the conduct of the Investigation at his/her Participating Site, and for protecting the rights, safety and well-being of the participants. As such the PI must ensure adequate supervision of the Investigation conduct at the Participating Site. If any tasks are delegated, the PI will maintain a log of appropriately qualified persons to whom he/she has delegated specified Investigationrelated duties. The PI will ensure that adequate training is provided and documented for all Investigation staff, prior to conducting assigned Investigation-related activities.

It is the CI’s responsibility to supervise the general conduct (e.g., study progress, communication, CIP training and support of the participating sites, annual reporting to the EC, end of Investigation notification(s) and results reporting…) of the Investigation. The CI fulfils both Investigator and Sponsor responsibilities, as outlined in ISO 14155:2020 and applicable regulations.

PI and CI shall each be referred to as «Investigator(s)».

This CIP (and its annexes) will be signed by each Participating Site and serve as contract between Sponsor and Participating Site in accordance with GCP (Section 1.17 Contract: “*A written, dated, and signed agreement between two or more involved parties that sets out any arrangements on delegation and distribution of tasks and obligations and, if appropriate, on financial matters. The protocol may serve as the basis of a contract.”* and Section 4.5.1: “[…] *The investigator/institution and the sponsor should sign the protocol, or an alternative contract, to confirm agreement.*”).

**CIP SYNOPSIS**

| Title of clinical Investigation («Investigation») | Evaluation of the implementation process of POCT-CRP devices in general practice in adults with cough. |
| --- | --- |
| CIP Short Title Acronym | FOD POCT CRP project |
| Sponsor name | KU Leuven |
| Coordinating Investigator | Prof Dr Jan Verbakel |
| Contact Address CI | Department of Public Health and Primary Care, KU Leuven Kapucijnenvoer 7, Blok J, 3000 Leuven, Belgium |
| Contact Email CI | jan.verbakel@kuleuven.be |
| Contact Phone CI | 016377286 |
| SIN number | Not applicable |
| Other public database number | Not applicable |
| Principal Investigators and Participating Sites | See list above of participating sites (page 2) |
| Medical condition or disease under investigation | Adults with acute cough |
| Study rationale | Evaluation of the implementation process (linkage, training and quality assurance) of three clinically validated POCT-CRP devices in general practice as part of standard of care, used in the FOD project ‘Evaluation on the organizational challenges of the implementation of POCT-CRP outside of hospital in adults with cough’ (NAPAMR 2020-2024-POCT), supervised by Prof. dr. Jan Verbakel (KU Leuven, Department of Public Health and Primary Care). |
| Primary objective | Linkage between clinically validated POCT-CRP devices in GP practices and the lab information system via middleware, after which the results can be medically validated by the clinical laboratory and re-structured in the GP's EMR through the calibrated channels |
| Secondary objective(s) | End-user training and quality assurance  Evaluation of the implementation process and stakeholder engagement |
| Clinical Investigation Design | Feasibility study and process evaluation of the implementation process of 3 Point-of-Care C-Reactive protein devices used within standard of care (CE label and within intended use):   - QuikRead go easy CRP test (Aidian Diagnostics, Espoo, Finland) - Cobas b101 (Roche Diagnostics, Mannheim, Germany) CRP test - Afinion 2 (Abbott, Oslo, Norway) CRP test |
| Endpoints | Connectivity (linkage) and themes from process evaluation |
| Sample Size | 27 GP practices sampling 5-15 adult patients each |
| Maximum duration of management of a Participant | 1 day |
| Maximum duration of the assessment | 5 minutes |
| Anticipate First Patient First Visit (FPFV) | 8^th^ September 2023 |
| Anticipate Last Patient Last Visit (LPLV) | 30^th^ November 2023 |
| Third Parties | Provision of devices and tests:   - Aidian Diagnostics, Espoo, Finland - Roche Diagnostics, Mannheim, Germany - Abbott, Oslo, Norway |

KU Leuven Protocol: FOD POCT CRP project

| Internal Ref.: S67992 | Version: 1.2_29Aug2023 |
| --- | --- |
|  |  |
| **STUDY FLOWCHART** |  |

Schedule of Events – Study specific Procedures / Assessments

| **Procedures/ Assessment** | **Screening** | **Testing and data collection** |
| --- | --- | --- |
| Visits / Contacts | Emergency department visit | |
| Visit Window (days) | 1 day | |
| Informed consent | X ^1^ |  |
| Inclusion / Exclusion  criteria | X |  |
| Use of point-of-care CRP test (1 of the selected 3 devices) |  | X |
| Reason for discontinuation | X | X |

*I : Informed Consent process should take place prior to all other study-related procedures at the screening visit*

CTC GEN-TMP-004-1.0_28Sep2022 Page 10 of 29 Protocol template for clinical investigation

Confidential & Proprietary with IMD, used within CE-label

**1 Background and Rationale**

One of the main causes of antimicrobial resistance (AMR) is the use of antibiotics in human medicine.^1,2^ Antibiotic resistance is an important and growing problem and has a lasting impact on our medical care in the coming decades. In the ambulatory care, most antibiotics are prescribed by general practitioners (GP).^3^ Although acute respiratory tract infections are generally self-limiting, Belgian GPs often write an antibiotic for it.^4^ Despite great efforts to prevent inappropriate antibiotic prescribing in primary care in Belgium, the objectives, as described in the National AMR Action Plan, are not yet achieved.^5^ Over the past decade, several interventions, such as point-of-care testing (POCT) were developed, launched and assessed, both in Belgium and in other (European) countries. But many of these interventions, including POCT, have not been widely implemented or evaluated in the Belgian context.^6^ Clinical studies in primary care (general practice) have demonstrated the use of CRP point-of-care testing (POCT) to direct antibiotic prescription resulting in a significant reduction of prescribing antibiotics without harming the patient. This intervention on adults with acute cough has also proven cost-effective, as well as effective in the longer term.^7,8^ In several European countries, POCT-CRP testing is part of the guidelines for good medical practice for acute cough/respiratory infections in general practice, for example in the Netherlands.^9^ Before POCT-CRP can be introduced nationally in Belgium, a number of organizational aspects need to be addressed.

**Work package 1: Link between point-of-care setting and clinical lab environment (connectivity with electronic patient record)**

An efficient supply chain is a fundamental requirement to ensure system reliability and reduce costs. Systems that can be applied to ensure the storage of test kits at the required temperature, guidelines that can control the correct disposal of test materials in a non-hazardous way and the impact on patient flow within the medical practice will be investigated. State of the art POCT hardware is equipped for patient and user identification, and for electronic data transfer of patient results to POCT middleware (already available in most hospital laboratories), to the laboratory information system (LIS) and to the electronic medical record (EMR). In this way, POCT results are made fully traceable in the LIS, available in the EMD and consultable via existing e-health platforms. The laboratory has expertise in establishing quality control of results and is familiar with the actions to be taken in case of discrepancies. The laboratory can provide support for technical problems (e.g. provision of back-up equipment) and can arrange contacts with manufacturers/distributors.

Point-of-care CRP Cartridges are for in vitro diagnostic use in the quantitative measurement of C-reactive protein (CRP) in human capillary (fingerstick) whole blood, and lithium-heparinized venous whole blood or plasma, using a point-of-care analyzer. The reagent cartridges are to be used by healthcare professionals at the point of care (POC) as well as in the clinical laboratory. The reagent cartridges are to be used as an aid in the decision to prescribe antibiotics in patients presenting with acute cough.

The three point-of-care CRP devices are manufactured in accordance with IVDD regulations. A single use cartridge is used for the analysis of C-reactive protein in capillary whole blood. The point-of-care CRP devices have embedded software and a supporting service software application.

The three clinically validated point-of-care CRP devices are prepared by the respective manufacturers and performance will be confirmed using liquid quality control. Transport of the devices and installation together with the initial training of the end-users (GPs and GP staff) will be performed by the manufacturers. The device (i.e. analyzer) should be operated between 5-27 degrees C. Individual cartridges can be used at ambient temperatures (18 – 26 degrees C). There is no manual preparation required. All packaging and labelling is in accordance with IVDD regulations.

To this end, we will install the clinically validated (i.e. clinical performance as part of standard of care has been established previously) POCT-CRP devices in 27 GP practices and provide the necessary IT support, whereby linkage between the device and the lab information system is possible via middleware (Roche Cobas Infinity POC), after which the results can be medically validated (i.e. final semi-automated check of results by clinical biologist) by the clinical laboratory and re-structured in the GP's EMR through the calibrated channels. For each participating central laboratory (Klinisch Laboratorium OLV Ziekenhuis Aalst, Medisch Labo Medina Aalter, LHUB-ULB, Brussel en Laboratoires Cliniques St-Luc, Brussel), a subset of practices (3 to 5 per central lab), will be provided with the middleware. All study-related activities (recruitment, informed consent, fingerprick blood sampling) will be performed at the GP practices by the participating GPs and their staff.

**Work package 2: End-user training and quality assurance**

There is evidence that uncontrolled use of POCT can have far-reaching negative consequences for both individuals and public health. One aspect will be to consider systems that can be put in place to ensure that all personnel performing POCT have received adequate training. This training will be provided by the manufacturer and clinical laboratory at a plenary meeting at the beginning of the implementation stage of this project. As user training is organised and monitored, the likelihood of pre-analytical errors is reduced and results will be more reliable. The laboratory and/or manufacturer will organise and certify training (state-of-the-art POCT equipment will only work if the user is identified and certified).

Quality assessment on a regular basis is a prerequisite so that the treating physician and the patient can have confidence in the test result. One should consider how quality can be assessed by recognised professional bodies or by the government itself. Clinical laboratories will be used to validate test results. Clinical laboratories have mandatory procedures for monitoring the quality of diagnostic tests that will be implemented in this pilot project (e.g. continuous monitoring of internal quality control, external quality control, distribution of reagents, technical support).

Performing internal quality controls (IQC) at regular intervals is key to ensuring that POCT instruments are functioning properly and that POCT reagents are providing accurate patient results. Quality control material target concentrations should be chosen so that one falls within the reference interval or close to decision limits of the assay and one in the abnormal range (high or low). For qualitative tests, one of the selected QC material should be negative and one positive. Imprecision as part of routine quality assurance will be evaluated in a subset of 15 practices (affiliated with the central lab of OLVZ Aalst) using a patient lithium heparin plasma pool (+/-20 mg/L) in addition to manufacturer specific internal quality control (iQC) material with low and high CRP concentration. Each iQC sample will be analysed at least 10 times per GP practice by the GP (staff), for each POCT-CRP device, spread over a period of 4 weeks each time. Imprecision will be evaluated according to CLSI EP05-A3. The criterion for acceptable imprecision is defined as a coefficient of variation (CV%) less than or equal to 10%.

Additionally, capillary blood samples will be prospectively collected from patients at the GP practices and will be immediately analyzed with the POCT-CRP device. All physicians and nurses from the GP practices will be informed about the study design. The POCT-CRP samples will be collected by the certified practice staff. In all patients from whom capillary samples will be collected, written informed consent will be obtained from patients by the GPs and/or their staff before sample collection during the study period (cfr. addendum 4.1). It is generally to be expected that GPs will use the POCT-CRP at least 20 times per device.

**Work package 3: Evaluation of the implementation process and stakeholder engagement**

Meeting stakeholder expectations can ensure that one achieves the intended results. Identifying the different stakeholders and their expectations is crucial and provides a strategy to involve them. For example, appointing an implementation champion can help accelerate the process of adaptation of POCTCRP, and national education campaigns can create the required awareness among patients to further embrace this new technology and support when (not) to use the test.

We will organise a survey of relevant stakeholders involved in this pilot project and the implementation of the POCT-CRP device using a survey and focus group discussions. From the experiences of the relevant stakeholders, we want to describe: how the implementation went in terms of organisation, evaluation of feasibility, feasibility, barriers and facilitators of implementation.

This study will use focus group discussions and surveys to gauge experiences with the POCT-CRP devices in general practice. Prior to the meeting, at pick-up of each device, all participants receive a survey that they can fill in immediately regarding the device used during the last period (up to 3 surveys per physician for up to 3 devices in total). The survey contains questions about the implementation of the POCT-CRP device in general practice, the feasibility of the procedure and the barriers and facilitators they faced during the implementation process. Scores will be used per question ranging from completely agree and agree to neutral, disagree and completely disagree. After completing the survey, a focus group discussion will be started. We will perform the discussion in person unless unforeseen circumstances prevent this. If necessary, the content and design of the focus group can be tested in advance with another physician who has experience in research. We are going to report the findings of the focus group discussion using the consolidated criteria for reporting qualitative research (COREQ) checklist.^10^

We will be recruiting GP practice staff (doctors and nurses) who will have used these devices in work package 1 and 2 to share their experiences. We strive to get a good distribution of staff from the various participating GP practices in the different focus groups.

We will organize 3 to 4 meetings with about 6 to 10 participants at each meeting. At the beginning of the meeting all participants will receive a document confirming their informed consent to participate in this study. After informed consent has been obtained, all participants get a survey that they can fill in immediately. It will take about 15 minutes to complete the survey. After all participants have completed the survey, the discussion will be started by the moderator of the focus group. The moderator will be one of the researchers, trained by the research team by means of a formal internal training in interviewing techniques as well as organizing a sham focus group to practice the different aspects of the focus group in realtime. We will make a video and audio-recording of the focus groups so that we can further analyze them afterwards. The focus group discussion will start with some general information about POCT-CRP devices in general practice provided by the moderator. The discussion will consist of open-ended questions listed in the interview guide which we also added to the addenda. The focus group discussion will take about 1 and a half hours.

The data analysis will be based on the QUAGOL guide for the analysis of qualitative data.^11^ After the first focus group is over, we will start with the analysis of the data. Every focus group discussion will be transcribed including the nonverbal signals and the interaction between the participants. At least two of the team members will read the transcript in detail to apprehend the essential features of the discussion. During this first stage of the QUAGOL criteria, the researcher will underline key phrases, which left an impression on the researcher. At the second stage, the researcher will make a narrative report which will give us an answer to the research questions. At least two researchers from the team will make narrative reports of the obtained data. The third stage consists of making concepts which reflect the essence of the data in response to the research question. The concepts are represented in a scheme, and if needed clarified. The most important concepts are highlighted. In stage four we look back at the data from the focus group discussions with the conceptual scheme in mind. We adapt, complete or refine the conceptual scheme with the information of the interview data that may have been overlooked. To optimize the conceptual schemes, we will organize a meeting with all researchers to compare and discuss the conceptual schemes. The concepts of the conceptual schemes are reviewed and compared with the conceptual scheme of the other focus group discussions in stage five so we can identify the most common concepts, themes and hypotheses. New themes, concepts or hypotheses are checked for their presence in previous focus group discussions so we can adapt their conceptual schemes. In stage six we can start with the actual coding process. We draw up a list of common concepts without any hierarchical order. The list will be discussed within the research team. When the list is completed, we will introduce the concepts as preliminary codes in the software program. In stage seven we read again the focus group discussions with the list of concepts at hand. Each significant passage of the focus group discussion has to be linked to one of the concepts of the list. If not, we need to adapt the list with concepts. The concept also must cover all significant ideas and messages from the focus group discussion. Stage eight consists of the analysis of the concepts linked to each relevant passage in the focus group discussions. The researcher tries to understand the specific meaning of these concepts. After the deeper analysis of the concepts, the researcher has a clear description of the concepts with their meaning, dimensions and characteristics. In stage nine, the researchers integrate all the concepts in a conceptual framework in response to the research question. The framework must describe all individual focus group discussions. At the final stage the researchers are able to describe the essential findings in answer to the research questions. It starts with a core category and related concepts. Significant quotes were added if necessary to understand the essence of the concepts.

**2 Objectives and Design**

# 2.1 Study objectives and hypotheses

Evaluation of the implementation process of three clinically validated POCT-CRP devices in general practice as part of standard of care, used in the FOD project ‘Evaluation on the organizational challenges of the implementation of POCT-CRP outside of hospital in adults with cough’ (NAPAMR 2020-2024POCT), supervised by Prof. dr. Jan Verbakel (KU Leuven, Department of Public Health and Primary Care).

# 2.2 Primary Endpoints

Connectivity (linkage) and themes from process evaluation

# 2.3 Secondary Endpoints

Not applicable

# 2.4 Design of the Clinical Investigation

Feasibility study and process evaluation

# 2.5 Justification for the design of the Investigation

This project is a feasibility and implementation project aiming to evaluate the feasibility of implementing point-of-care CRP testing in primary care for patients with acute cough

# 2.6 Expected Duration of the Investigation

The expected overall investigation duration will be from first patient first visit until last patient first visit. We expect to achieve the target number of cases in 3 months after first patient first visit.

For a single patient the expected investigation is from informed consent after first contact with the GP until the end of that consultation.

The end of the Investigation will be notified by the Sponsor within 15 days of the end of the Investigation to the EC.

**3 Eligibility Criteria**

# 3.1 Inclusion criteria

For patients:

Patients eligible for inclusion in this Investigation have to meet **all** of the following criteria:

1. Written informed consent must be obtained prior to any screening procedures
2. Age >18 years
3. Presenting to the general practitioner with acute cough
4. Treating general practitioner judges patient is eligible to test CRP level by means of the point-ofcare test as per usual care and current guidelines on patients with acute cough.

All participants that are considered for Investigation participation per the above criteria, will be documented on the Screening Log, including Screen Failures. For stakeholders taking part in the survey and focus group discussion:

GP staff (doctors, nurses, medical staff), clinical biologists, device manufacturers and patients’ representatives participating in this study of the evaluation of the implementation process will be invited to take part in the survey and focus group discussion. The stakeholders will have to meet **all** of the following criteria to be eligible for inclusion:

1. Written informed consent must be obtained prior to any screening procedures
2. Age >18 years
3. Experience during the evaluation of the implementation process with the conduct of a point-ofcare CRP test in ambulatory care

# 3.2 Exclusion criteria

Participants (both patients and stakeholders) eligible for this Investigation must **not** meet any of the following criteria:

1. Unable to provide written informed consent
2. Clinically unstable participants, requiring immediate treatment.
3. Age <18 years

Participants who meet one or more of the above exclusion criteria **must not proceed** to be enrolled/randomized in the Investigation and will be identified on the Screening Log as Screen Failure.

**4 Study Procedures**

# 4.1 Participant consent and withdrawal of consent

The Investigation will be conducted only on the basis of prior informed consent by the participants and/or their legally authorized representative(s). As such, no Investigation-related procedures will be conducted prior to obtaining written informed consent from potential participants.

The process for obtaining and documenting initial and continued informed consent from potential participants will be conducted in accordance with ISO 14155:2020, applicable regulatory requirements and internal Standard Operating Procedures (SOPs).

All originally signed obtained Informed Consent Forms (ICFs) must be retained/archived in the ISF at the Participating Site and must not be destroyed (even when a scanned copy is available) before expiration of the legal archiving term as defined in the CIP section entitled “Archiving”.

Participants may voluntarily withdraw consent to participate in the Investigation for any reason at any time. The participant’s request to withdraw from the Investigation must always be respected without prejudice or consequence to further treatment. Consent withdrawal will be documented in the participant’s medical record.

Study data and samples collected before withdrawal can be used in the study. No new study data or samples will be collected after withdrawal of the participant.

# 4.2 Selection of Participants / Recruitment

All adults patients presenting to the participating general practitioners with acute cough and within the eligibility criteria will be asked to participate in the study. Written informed consent will be obtained from patients by the GPs and/or their staff.

All adult stakeholders taking part in the study on the evaluation of the implementation process will be asked to participate in the survey and focus group discussions.

# 4.3 Study specific procedures

In patients with acute cough, a point-of-care CRP test will be performed as per international guidelines and according to the judgement of the general practitioner.

# 4.4 Premature discontinuation

Participants may voluntarily discontinue and/or prematurely end their participation in the Investigation for any reason at any time. In such case the Investigator must make a reasonable effort to contact the participant (e.g., via telephone, e-mail, letter) in order to document the primary reason for this decision.

Similarly, the Sponsor, EC or authorized regulatory authority can decide to halt or prematurely terminate the Investigation when new information becomes available whereby the rights, safety and well-being of participants can no longer be assured, when de integrity of the Investigation has been compromised, or when the scientific value of the Investigation becomes obsolete and/or unjustifiable. In case the Sponsor decides to temporary halt or prematurely end the Investigation, or to close a Participating Site in case of major non-compliance and/or critical safety issues, the Sponsor will notify the concerned EC within 15 days of early termination or temporary halt, providing a justification of the event. In the event that the sponsor has temporarily halted or early terminated the Investigation on safety grounds, the Sponsor will inform the EC within 24 hours of the event. In absence of EUDAMED, local guidelines with regards to notifications and submissions to the concerned EC will be followed.

Circumstances requiring premature treatment interruption or discontinuation of the Investigation, include but are not limited to:

• Investigation participation while in violation of the inclusion and/or exclusion criteria

In any such case of early termination, the Investigator will continue to closely monitor the participant’s condition and ensure adequate medical care and follow-up. It is recommended that follow-up information will be collected as follows:

During our feasibility study, routine medical standard of care will take place as usual by the treating physician. Medical care and follow-up will be provided by the treating physician. Participants will not be in direct contact with the clinically validated diagnostic device. All relevant information on adverse events will be collected by contacting the GP.

For participants whose status is unclear because they fail to appear for visits without stating an intention to discontinue or withdraw, the Investigator must make every effort to demonstrate “due diligence" by documenting in the source documents which steps have been taken to contact the participant to clarify their willingness and ability to continue their participation in the Investigation (e.g., dates of telephone calls, registered letters, etc.).

A participant should not be considered lost to follow-up until due diligence has been completed.

**5 Statistics and Data Analysis**

Statistical analysis will be performed in accordance with ICH E9; a detailed description of the analysis is provided in the study-specific Statistical Analysis Plan (SAP). ICH E3 and E8 will guide the structure and content of the clinical study report.

We will organise a survey of relevant stakeholders involved in this pilot project and the implementation of the POCT-CRP device using a survey and focus group discussions. From the experiences of the relevant stakeholders, we want to describe: how the implementation went in terms of organisation, evaluation of feasibility, feasibility, barriers and facilitators of implementation.

This study will use focus group discussions and surveys to gauge experiences with the POCT-CRP devices in general practice. At the beginning of the meeting, all participants receive a survey that they can fill in immediately. The survey contains questions about the implementation of the POCT-CRP device in general practice, the feasibility of the procedure and the barriers and facilitators they faced during the implementation process. Scores will be used per question ranging from completely agree and agree to neutral, disagree and completely disagree. After completing the survey, a focus group discussion will be started. We will perform the discussion in person unless unforeseen circumstances prevent this. If necessary, the content and design of the focus group can be tested in advance with another physician who has experience in research. We are going to report the findings of the focus group discussion using the consolidated criteria for reporting qualitative research (COREQ) checklist.^10^

# 5.1 Sample Size Determination

Approximately 27 GP practices recruiting 5-15 adult patients per practice will take part in the first part of the study (WP1 and WP2). Given this project is a feasibility on the implementation study and the qualitative nature of the process evaluation, no formal sample size calculation is applicable. For WP3 (survey and focus group discussions), all stakeholders involved (doctors, nurses, medical staff, clinical biologists, device manufacturers and patients’ representatives) will be invited to participate. We will perform focus group discussions until theoretical saturation is reached. We will include 5 to 10 participants per group. The exact number of sessions cannot be determined in advance, but based on previous research we expect to perform three to four interviews.

# 5.2 Statistical Analysis

For work package 1 and 2 only descriptive analyses will be performed describing the baseline characteristics of the participating GP practices. Given the experiences by the participants and evaluation of the implementation process will be the subject of work package 3, no predefined hypotheses will be tested in work package 1 and 2.

The data analysis of work package 3 (focus group discussions) will be based on the QUAGOL guide for the analysis of qualitative data.^11^ After the first focus group is over, we will start with the analysis of the data. Every focus group discussion will be transcribed including the nonverbal signals and the interaction between the participants. At least two of the team members will read the transcript in detail to apprehend the essential features of the discussion. During this first stage of the QUAGOL criteria, the researcher will underline key phrases, which left an impression on the researcher. At the second stage, the researcher will make a narrative report which will give us an answer to the research questions. At least two researchers from the team will make narrative reports of the obtained data. The third stage consists of making concepts which reflect the essence of the data in response to the research question. The concepts are represented in a scheme, and if needed clarified. The most important concepts are highlighted. In stage four we look back at the data from the focus group discussions with the conceptual scheme in mind. We adapt, complete or refine the conceptual scheme with the information of the interview data that may have been overlooked. To optimize the conceptual schemes, we will organize a meeting with all researchers to compare and discuss the conceptual schemes. The concepts of the conceptual schemes are reviewed and compared with the conceptual scheme of the other focus group discussions in stage five so we can identify the most common concepts, themes and hypotheses. New themes, concepts or hypotheses are checked for their presence in previous focus group discussions so we can adapt their conceptual schemes. In stage six we can start with the actual coding process. We draw up a list of common concepts without any hierarchical order. The list will be discussed within the research team. When the list is completed, we will introduce the concepts as preliminary codes in the software program. In stage seven we read again the focus group discussions with the list of concepts at hand. Each significant passage of the focus group discussion has to be linked to one of the concepts of the list. If not, we need to adapt the list with concepts. The concept also must cover all significant ideas and messages from the focus group discussion. Stage eight consists of the analysis of the concepts linked to each relevant passage in the focus group discussions. The researcher tries to understand the specific meaning of these concepts. After the deeper analysis of the concepts, the researcher has a clear description of the concepts with their meaning, dimensions and characteristics. In stage nine, the researchers integrate all the concepts in a conceptual framework in response to the research question. The framework must describe all individual focus group discussions. At the final stage the researchers are able to describe the essential findings in answer to the research questions. It starts with a core category and related concepts. Significant quotes were added if necessary to understand the essence of the concepts.

## 5.2.1 Efficacy Analysis

| **Endpoint** | **Statistical Analysis Methods** |
| --- | --- |
| Primary | Connectivity (linkage) and themes from process evaluation |

## 5.2.2 Other Analysis

Not applicable.

# 5.3 Interim Analysis and Final Database Lock

No interim analyses apart from the thematic analyses of the focus groups are planned.

**6 Data handling**

Data handling and data flows for the Investigation are summarized below and will be described in more detail in the Investigation-specific Data Management Plan (DMP).

Data will be gathered in an electronic CRF, Castor EDC (Castor, New York, USA).

Data will be pseudonymised. After enrolment and informed consent an ID code will be appointed to each participant.

We will gather the results of the three point-of-care CRP device measurements.

# 6.1 Data Collection Tools and Source Document Identification

## 6.1.1 Operational aspects

Data collection, handling, processing and transfer for the purpose of this Investigation will be performed in compliance with applicable regulations, guidelines for clinical trials and internal procedures, as follows:

*6.1.1.1 Data collection*

No specific source data will be collected during work package 1 and work package 2 of this study.

For the collection and storage of data in work package 3 (survey and focus group discussions), we will use OneDrive for Business, provided by KU Leuven.

*6.1.1.2 Data Validation*

All data as part of work package 3 relating to the Investigation must be prepared and validated by the Investigator.

*6.1.1.3 Data Management and data transfer*

All data as part of work package 3 will be treated confidentially and with due care during the project. Only relevant information will be collected and this will be limited to what is necessary for the purposes of the research question. The information obtained through the research will be collected in a structured manner in a secured computer document. If possible, this file will be stored in a project- or team-based storage location on a KU Leuven or UZ Leuven server, in OneDrive for Business, provided by KU Leuven. Otherwise, a personal computer secured by password, two-step verification or specialized program such as 7-Zip or AEScrypt will be used.

No data or results will be made available to the participating device manufacturers during this study. Final results as part of a study report will be made available to all interested parties after publication as part of the requirements provided by FOD Volksgezondheid.

Data allowing identification of participants will be pseudonymized. This means that personal data will be processed in such a way that it can no longer be attributed to a specific person without the use of additional information. However, the direct link between the data subject and the dataset will not be broken, but will be encoded using a code. This code contains neither the name, nor initials, nor elements that could lead to identification (e.g., date of birth or place of birth). The codes will be kept in a separate and encrypted file, only in the possession of the supervisor(s) and the students involved. This file will not be kept on the same laptop or desktop as the data files.

Anything said by the participants during the focus groups will be recorded via audio recordings. The audio recordings will only be listened to and transcribed by the researchers. Immediately after transcription, the audio recordings, the diary entries, the interviewer's personal notes will be destroyed. Transcripts of the interviews will not include names or any other data that will allow identification. In the actual master's thesis, some excerpts from the interviews will be quoted in an anonymous manner and in such a way that it is not possible to trace the statement back to a particular participant. The transcripts will be kept in a secure computer document that is only in the possession of the supervisor(s) and the students involved. If possible, this file will be kept in a project- or team-based storage location on a KU Leuven or UZ Leuven server, in OneDrive for Business, offered by KU Leuven. Otherwise, a personal computer secured by password, two-step verification or specialized program such as 7-Zip or AEScrypt is used. GROUP BIOMEDICAL SCIENCE HERESTRAAT 49, O&N II - BUS 700 BE-3000 LEUVEN 2020.10 When sending a copy of confidential information or documents, they will always be encrypted with a password or unique code. For sending large files, Belnet Filesender will be used. Applications such as Google Drive, a personal OneDrive (with the exception of OneDrive for Business, offered by KU Leuven) or WeTransfer will be avoided at all times. Upon completion of the project, files will be delivered to the supervisor who will further ensure their safe keeping. In accordance with KU Leuven policy, these files will be kept for 10 years, after which it will be reassessed whether it is considered useful to keep the data any longer.

# 6.2 Archiving

As specified in ISO 14155:2020 section 8.6, the Sponsor and Investigator/Participating Site will maintain a record of the location(s) of all respective Essential Clinical Investigation Documents. The Sponsor should ensure that the Investigator has control of and continuous access to the data reported to the Sponsor during the Investigation.

The Investigator/Participating Site should have control of all Essential Documents and records generated by the Investigator/Participating Site before, during and following termination of the Investigation.

The Sponsor and Investigator is responsible for archiving study specific documentation, according to ISO 14155:2020. Source data and site-specific study documents (such as but not limited to the original signed ICFs) will be archived by the Participating Site(s) according to local practice, and for a period of at least 10 years after the clinical Investigation with the device in question has ended, or, in the event that the device is subsequently placed on the market, at least 10 years after the last device has been placed on the market.^^[[1]](#footnote-2)^^ Archived data may be held on electronic record, provided that media back-up exists, hard copies can be obtained, if required and measures are taken to prevent accidental or premature loss or destruction of data. Destruction of Essential Documents prior to, during or upon completion of the required archival period, will require written authorisation from the Sponsor.

**7 Ethical and Regulatory Considerations**

# 7.1 Ethics Committee (EC) review & reports

Before the start of the Investigation, this CIP and other related documents (e.g., ICF, IB, etc.) will be submitted for review to the EC for authorization. The Investigation shall not commence until such approval have been obtained.

It is the responsibility of the CI to produce the APR and submit to the EC within 30 days of the anniversary date on which favourable opinion to start the Investigation was given, and annually until the Investigation is declared ended.

The CI must notify the EC of the end of the Investigation. This notification must be made within 15 days of the end of the Investigation. For multinational investigations, local guidelines with regards to end of trial notification will be followed. The Sponsor must notify the EC in case of a temporary halt or early termination of the Investigation. This notification must be made within 15 days of the temporary halt or

early termination, providing a justification of the event. In the event that the Sponsor has temporarily halted or early terminated the Investigation on safety grounds, the EC must be informed within 24 hours of the event.

The CI will submit a final report with the results of the Investigation, including any publications/abstracts, to the EC within 1 year of Investigation termination, or within 6 months for paediatric Investigations. In case of a temporary halt or early termination this report must be provided within 3 months.

# 7.2 Regulatory Compliance

The Investigation will be conducted in compliance with the most recent version of the Declaration of Helsinki, and with the GDPR, the relevant Belgian laws implementing the GDPR, the Belgian Law of August 22^nd^, 2002 on patient rights, and all other applicable legal and regulatory requirements.

# 7.3 CIP / GCP compliance

The Investigation must be performed in accordance with the CIP, ISO 14155:2020, and applicable regulatory and country-specific requirements. ISO 14155:2020 is an international ethical and scientific quality standard for designing, conducting, recording and reporting clinical investigations that involve the participation of human participants. Compliance with this standard provides public assurance that the rights, safety and well-being of participants are protected, consistent with the principles that originated in the Declaration of Helsinki, and that the study data are credible, reliable and reproducible.

The Investigator and study team acknowledge and agree that prospective, planned deviations or waivers to the CIP are not permitted under applicable regulations on clinical investigations. However, should there be an accidental CIP- deviation, such deviation shall be adequately documented in the source documents and on the relevant forms and reported to the CI and Sponsor. Deviations should also be reported to the EC as part of the EC’s continued review of the Investigation (e.g., through the ASR, APR, etc.). CIP deviations which are found to frequently recur, will require (immediate) action. The Investigator acknowledges that such recurring CIP deviations could potentially be classified as a serious violation of ICH and/or the CIP.

It is understood that “a serious violation” is likely to affect to a significant degree:

- the safety or physical or mental integrity of the participants; or
- the scientific validity of the Investigation

The Investigator is expected to take any immediate action required to protect the safety of any participant included in the Investigation, even if this action represents a deviation from the CIP. In such cases, the Sponsor should be notified of this action and the EC should be informed according to local procedures and regulations.

# 7.4 Data protection and participant confidentiality

The Investigation will be conducted in compliance with the requirements of the GDPR, the relevant Belgian laws implementing the GDPR, including the Belgian Privacy Act of July 30^th^, 2018 on the protection of privacy in relation to the processing of personal data. Any collection, processing and disclosure of personal data, such as participant health and medical information is subject to compliance with the aforementioned personal data protection laws (cfr. Data Processing Annex (DPA) in Appendix 2). No personal data may be transferred outside the European Economic Area.

Any personal data shall be treated as confidential at all times including during collection, handling and use or processing, and the personal data (including in any electronic format) shall be stored securely at all times and with all technical and organizational security measures that would be necessary for compliance with EU and national data protection legislation (whichever is more stringent). The Sponsor shall take appropriate measures to ensure the security of all personal data and guard against unauthorized access thereto or disclosure thereof or loss or destruction while in its custody.

# 7.5 Amendments

As specified in ISO 14155:2020 section 7.5.1, amendments must not be implemented prior to EC review and/or approval, as applicable. Under emergency circumstances, deviations from the CIP to protect the rights, safety or well-being of human subjects may proceed without prior approval of the sponsor and the EC. Such deviations shall be documented and reported to EC as soon as possible.

Amendments to the Investigation are regarded as ‘substantial’ when they are likely to have a substantial impact on the safety, health or rights of the subjects or on the robustness or reliability of the clinical data generated by the investigation.

# 7.6 Post-Study activities

No post-study activities are applicable.

**8 Research Registration, Dissemination of Results and Publication Policy**

The Declaration of Helsinki (most recent version) and European and Belgian regulations require that every research involving human participants be registered in a publicly accessible database before recruitment of the first participant. The CI is responsible for registering the Study.

In addition, the CI will fulfil its ethical obligation to disseminate and make the research results publicly available. As such, the CI is accountable for the timeliness, completeness and accuracy of the reports. Researchers, authors, sponsors, editors and publishers must adhere to accepted guidelines for ethical reporting. Negative and inconclusive, as well as positive results, must be published or otherwise made publicly available. Sources of funding, institutional affiliations and conflicts of interest must be declared in publication.

Publications will be coordinated by the CI. Authorship to publications will be determined in accordance with the requirements published by the International Committee of Medical Journal Editors, and in accordance with the requirements of the respective medical journal.

For multicentre Investigations, it is anticipated that the primary results of the overall Investigation shall be published in a multicentre publication.

Participating Sites are not allowed to publish any subset data or results from the Investigation prior to such multicentre publication.

Any publication by a Participating Site must be submitted to the Sponsor for review at least 30 calendar days prior to submission or disclosure. Sponsor shall have the right to delay the projected publication for a period of up to 3 months from the date of first submission to the Sponsor in order to enable the Sponsor to take steps to protect its intellectual property rights and know-how.

1. **Intellectual Property**

Any know how, inventions, methods, developments, innovations, discoveries and therapies, whether patentable or not, arising from the Investigation or made in the performance of the CIP (“Inventions”) shall vest in the Sponsor. If the Investigation is multicentric, the Participating Site, its employees and Investigator(s) shall promptly disclose to the Sponsor any such Inventions. The Sponsor and the Participating Site have expressly agreed that any and all study data as collected and prepared in the performance of the CIP shall be the sole property of the Sponsor.

Sponsor will deliver a report to FOD Volksgezondheid containing *conclusions* and *lessons learned* (evaluation of the use of POCT, training of users, budget implications, etc.) from the study. However, no study data will be contained in the report.

1. **Joint Commission International (JCI)**

In order to ensure the same quality and safety standards in patient care for clinical research as commonly applied by the Sponsor in its regular activities, and in accordance with JCI standards, the Sponsor shall comply with the following obligations: (a) the Sponsor will use trained and qualified employees or contractors to manage and coordinate the Investigation; (b) the Sponsor will ensure that multi-center Investigation reporting is reliable and valid, statistically accurate, ethical, and unbiased. (c) the Sponsor will not grant incentives, other than standard compensations and reimbursement of costs, to participants or to Participating Site’s staff that would compromise the integrity of the research; (d) the Sponsor is responsible for monitoring and evaluating the quality, safety, and ethics of the Investigation and will respect the participating site’s policies and processes when performing such monitoring and evaluation activities; (e) the Sponsor will protect the privacy and confidentiality of the participants in accordance with all applicable laws.

1. **References**
2. Malhotra-Kumar S, Lammens C, Coenen S, et al. Effect of azithromycin and clarithromycin therapy on pharyngeal carriage of macrolide-resistant streptococci in healthy volunteers: a randomised, double-blind, placebo-controlled study. The Lancet 2007;369(9560):482-90.
3. Malhotra-Kumar S, Van Heirstraeten L, Coenen S, et al. Impact of amoxicillin therapy on resistance selection in patients with community-acquired lower respiratory tract infections: a randomized, placebocontrolled study. Journal of Antimicrobial Chemotherapy 2016;71(11):3258-67.
4. Riziv. Farmanet. <https://www.riziv.fgov.be/nl/statistieken/geneesmiddel/Paginas/geneesmiddelengroepvoorschrijvers. aspx>.
5. Bruyndonckx R, Adriaenssens N, Versporten A, et al. Consumption of antibiotics in the community, European Union/European Economic Area, 1997–2017. Journal of Antimicrobial Chemotherapy 2021;76(Supplement_2):ii7-ii13.
6. AMR: Operationeel plan. [https://www.health.belgium.be/nl/amr-operationeel-plan.](https://www.health.belgium.be/nl/amr-operationeel-plan)
7. Leroy R, Christiaens W, Maertens de Noordhout C, et al. Voorstellen voor een effectiever antibioticabeleid in België-Synthese. Health Services Research (HSR) Brussel: Federaal Kenniscentrum voor de Gezondheidszorg (KCE) 2019.
8. Little P, Stuart B, Francis N, et al. Effects of internet-based training on antibiotic prescribing rates for acute respiratory-tract infections: a multinational, cluster, randomised, factorial, controlled trial. The Lancet 2013;382(9899):1175-82.
9. Oppong R, Jit M, Smith RD, et al. Cost-effectiveness of point-of-care C-reactive protein testing to inform antibiotic prescribing decisions. Br J Gen Pract 2013;63(612):e465-71.
10. Verheij T, Hopstaken RM, Prins JM, et al. NHG-Standaard Acuut hoesten. Huisarts & Wetenschap 2011;54(2):86-92.
11. Tong A, Sainsbury P, Craig J. Consolidated criteria for reporting qualitative research (COREQ): a 32item checklist for interviews and focus groups. International Journal for Quality in Health Care. 2007;19(6):349-57.
12. Dierckx de Casterlé B, Gastmans C, Bryon E, Denier Y. QUAGOL: A guide for qualitative data analysis. International Journal of Nursing Studies. 2012;49:360-371.

**Appendices**

*Additional documents can be included as Appendix, such as ICF, PRO’s, questionaires, scores, specific study information, additional guidelines (e.g., coding, classification) and scoring information, etc.*

| KU Leuven Protocol: FOD POCT CRP project Internal Ref.: S67992 Version: 1.2_29Aug2023 | |  |
| --- | --- | --- |
| **12 Appendix 1: Clinical Investigation Plan history** | |  |
|  | *Complete the table below for each CIP amendment, provide a summary of the modifications made to the CIP while referencing the relevant CIP*  *complete the table for each subsequent CIP amendment, while maintaining the completed table for previous*  *CIP amendments have been issued: keep the amendment table, but do not complete.* | *sections that were altered. Copy, insert*  *CIP amendments.* |
| *and*  *If no* |  |  |

| **Original CTP version:** | 1.0 dated 24/08/2023 |
| --- | --- |

| **Amendment #1:** | <CIP version number> dated 24/08/2023 |
| --- | --- |
|  | Modifications made / Reason for amendment: |
| <CIP section reference> | <describe modifications made> |
| < CIP section reference> | <describe modifications made> |
| < CIP section reference> | <describe modifications made> |
| < CIP section reference> | <describe modifications made> |

| **Amendment #2:** | <CIP version number> dated 24/08/2023 |
| --- | --- |
|  | Modifications made / Reason for amendment: |
| < CIP section reference> | <describe modifications made> |
| < CIP section reference> | <describe modifications made> |
| < CIP section reference> | <describe modifications made> |
| < CIP section reference> | <describe modifications made> |

CTC GEN-TMP-004-1.0_28Sep2022 Page 24 of 29 Protocol template for clinical investigation

Confidential & Proprietary with IMD, used within CE-label

Internal Ref.: S67992

**13 Appendix 2: Data Processing Annex (DPA) between Sponsor and Participating Site(s)**

*Only applicable in case of a multicentric Investigation. For a monocentric Investigation at UZ Leuven this appendix can be deleted.*

Definitions:

- “CIP” means the document entitled “Evaluation of the implementation process of POCT-CRP devices in general practice in adults with cough” with reference s67992 containing the details of the academic Investigation as developed by the Sponsor and approved by the relevant EC/CA as applicable.
- “Sponsor” means University Hospitals Leuven (UZ Leuven).
- Participating site acts as a data processor as defined under article 4, 8) of the Regulation (EU) 2016/679 (“Data Processor”) for the Sponsor who acts as data controller as defined under article 4, 7) of the Regulation (EU) 2016/679 (“Data Controller”).
- “Applicable Law” means any applicable data protection or privacy laws, including:
  - 1. the Regulation (EU) 2016/679 also referred as the General Data Protection Regulation ("GDPR");
    2. other applicable laws that are similar or equivalent to or that are intended to or implement the laws that are identified in (a) of this definition;
- "Personal Data" means any information relating to an identified or identifiable natural person (”Data Participant”), including without limitation pseudonymized information, as defined in Applicable Law and described in the CIP.

Rights and obligations:

1. The Data Processor is instructed to process the Personal Data for the term of the Investigation and only for the purposes of providing the data processing tasks set out in the CIP. The Data Processor may not process or use Personal Data for any purpose other than a Data Participant’s medical records, or other than provided in the instructions of the CIP, including with regard to transfers of personal data to a third country or an international organization, unless the Data Processor is required to do so according to Union or Member State law.
2. Data Processor shall at all times maintain a record of processing of Personal Data in accordance with Applicable Law and if the Data Processor considers an instruction from the Data Controller to be in violation of the Applicable Law, the Data Processor shall promptly inform the Data Controller in writing about this.
3. The Data Processor must ensure that persons authorized to process the Personal Data have committed themselves to confidentiality or are under an appropriate statutory obligation of confidentiality.
4. The Data Processor shall implement appropriate technical and organizational measures to prevent that the Personal Data processed is:
   1. accidentally or unlawfully destroyed, lost or altered,
   2. disclosed or made available without authorization, or (iii) otherwise processed in violation of Applicable Law.

5. The appropriate technical and organizational security measures must be determined with due regard for:

1. the current state of the art,

-TMP-004-1.0_28Sep2022 Page 25 of 29 Protocol template for clinical investigation

Confidential & Proprietary h IMD, used within CE-label

1. the cost of their implementation, and
2. the nature, scope, context and purposes of processing as well as the risk of varying likelihood and severity for the rights and freedoms of natural persons.
3. Taking into account the nature of the processing, the Data Processor shall assist the Data Controller, by means of appropriate technical and organizational measures, insofar as this is possible, in fulfilling its obligation to respond to requests from Data Participants pursuant to laws and regulations in the area of privacy and data protection (such as, the right of access, the right to rectification, the right to erasure, the right to restrict the processing, the right to data portability and the right to object)
4. The Data Processor shall upon request provide the Data Controller with sufficient information to enable the Data Controller to ensure that the Data Processor's obligations under this DPA are complied with, including ensuring that the appropriate technical and organizational security measures have been implemented.
5. The Data Controller is entitled to appoint at its own cost an independent expert, reasonably acceptable to the Data Processor, who shall have access to the Data Processor's data processing facilities and receive the necessary information for the sole purpose of auditing whether the Data Processor has implemented and maintained said technical and organizational security measures. The expert shall upon the Data Processor's request sign a non-disclosure agreement provided by the Data Processor, and treat all information obtained or received from the Data Processor confidentially, and may only pass on, after conferral with the Data Processor, the findings as described under 10) (ii) below to the Data Controller.
6. The Data Processor must give authorities who by Union or Member State law have a right to enter the Data Controller's or the Data Controller's processors’ facilities, or representatives of the authorities, access to the Data Processor's physical facilities against proper proof of identity and mandate, during normal business hours and upon reasonable prior written notice.
7. The Data Processor must without undue delay in writing notify the Data Controller about:
   1. any request for disclosure of Personal Data processed under the CIP by authorities, unless expressly prohibited under Union or Member State law,
   2. any finding of (a) breach of security that results in accidental or unlawful destruction, loss, alteration, unauthorized disclosure of, or access to, Personal Data transmitted, stored or otherwise processed by the Data Processor under the CIP, or (b) other failure to comply with the Data Processor's obligations, or
   3. any request for access to the Personal Data (with the exception of medical records for which the Data Processor is considered data controller) received directly from the Data Participants or from third parties.
8. Such a notification from the Data Processor to the Data Controller with regard to a breach of security as meant in 10) (ii)(a) above will contain at least the following information:
   1. the nature of the Personal Data breach, stating the categories and (by approximation) the number of Data Participants concerned, and stating the categories and (by approximation) the number of the personal data registers affected (datasets);
   2. the likely consequences of the Personal Data breach;
   3. a proposal for measures to be taken to address the Personal Data breach, including (where appropriate) measures to mitigate any possible adverse effects of such breach.
9. The Data Processor shall document (and shall keep such documentation available for the Data Controller) any Personal Data breaches, including the facts related to the Personal Data breach, its effects and the corrective measures taken. After consulting with the Data Controller, the Data Processor shall take any measures needed to limit the (possible) adverse effects of Personal Data breaches (unless such consultation cannot be awaited due to the nature of the Personal Data breach).
10. The Data Processor must promptly and reasonably assist the Data Controller (with the handling of (a) responses to any breach of security as described in 10) (ii) above and (b) any requests from Data Participants under Chapter III of the GDPR, including requests for access, rectification, blocking or deletion. The Data Processor must also reasonably assist the Data Controller by implementing appropriate technical and organizational measures for the fulfilment of the Data Controller's obligation to respond to such requests.
11. The Data Processor must reasonably assist the Data Controller with meeting the other obligations that may be incumbent on the Data Controller according to Union or Member State law where the assistance of the Data Processor is implied, and where the assistance of the Data Processor is necessary for the Data Controller to comply with its obligations. This includes, but is not limited to, at the request to provide the Data Controller with all necessary information about an incident under 10) (ii), and all necessary information for an impact assessment in accordance with Article 35 and Article 36 of the GDPR.

Subprocessor:

1. The Data Processor may only engage a subprocessor, with prior specific or general written consent from the Data Controller. The Data Processor undertakes to inform the Data Controller of any intended changes concerning the addition or replacement of a subprocessor by providing a reasonable prior written notice to the Data Controller. The Data Controller may reasonably and in a duly substantiated manner object to the use of a subprocessor. The Data Processor must inform the Data Controller in writing of the discontinued use of a subprocessor.
2. Prior to the engagement of a subprocessor, the Data Processor shall conclude a written agreement with the subprocessor, in which at least the same data protection obligations as set out in this DPA shall be imposed on the subprocessor, including obligations to implement appropriate technical and organizational measures and to ensure that the transfer of Personal Data is done in such a manner that the processing will meet the requirements of the Applicable Law.
3. The Data Controller has the right to receive a copy of the relevant provisions of Data Processor's agreement with the subprocessor related to data protection obligations. The Data Processor shall remain fully liable to the Data Controller for the performance of the subprocessor obligations under this DPA. The fact that the Data Controller has given consent to the Data Processor's use of a subprocessor is without prejudice for the Data Processor's duty to comply with this DPA.

**14 Appendix 3: Financial Information Participating Site**

Sponsor, for the purposes of this Exhibit 2 represented by KU Leuven Research & Development, with offices at Waaistraat 6, 3000 Leuven, Belgium, VAT number BE 0419.052.173 (hereinafter “KU Leuven Research & Development”), acting on the request of prof. dr. Jan Verbakel has been awarded a assignment by the FOD Volksgezondheid and will use part of the budget for the purpose of paying each Participating Site for their assistance in performing the assignment:

**A fixed fee of 100,00 EUR**

**All fees are overhead included and VAT included**

Participating Site shall send invoices to :

KU Leuven Research & Development

F.a.o. Invoicing LRD

KU Leuven Research & Development

Waaistraat 6, box 5105 3000 Leuven, Belgium

lrd.efacturen@kuleuven.be (PDF-format)

Invoice(s) shall be issued at the end of the Study in conformity with the following KU LEUVEN requirements:

1. Participating Site’s name and address;
2. a detailed description and breakdown of the services and the date(s) of performance of the services;
3. Participating Site’s bank account details including IBAN & Swift Code;
4. Participating Site’s VAT number (if applicable);
5. KU LEUVEN’s VAT number BE0419.052.173;
6. the net amount payable and the VAT percentage or exemption of VAT (with disclosure relating to the clause of exemption);
7. the purchase order number.

**Participating Site’s bank details :**

Bank Details

Payments are to be made to: ….. Account number: ….. Name Accountholder: ….. Address accountholder: ….. Name & address of the bank: ….. BIC/SWIFT code: ….. IBAN number: …..

Communication/reference: …..

**On behalf of the Participating Site** Signature:

Name: […]

Title: GP

Date: …………………….

**On behalf of KU Leuven R&D**

Signature:

Name: Paul Van Dun

Title: General Manager

Date: …………………….

Name: Sabien Vanlangendonck

Title: Head Flemish and Federal Government Funding

Date: …………………….

For internal approval Signature:

Name: prof. dr. Jan Verbakel

Title: Principal Investigator

Date: …………………….

# III. Interview guide

- How did you experience the implementation of the POCT CRP device in your practice?
- What are the advantages of having a POCT CRP available for a patient with acute cough?
- What are the disadvantages of having a POCT CRP available for a patient with acute cough?
- How was the implementation of the POCT CRP device organized in your practice?
  - How did the installation of the device go?
  - Did you encounter any technical difficulties while using the device? If yes, which ones? How did you address them?
  - How did the integration of the device with the electronic medical record go?
  - Who was the point of contact in your practice for problems or questions regarding the POCT CRP device?
- How does the use of the POCT CRP device fit into your workflow?
  - For which patients did you use the POCT CRP device, and for which you did not?
  - What made you decide to use the device?
  - What made you decide not to use the device?
  - At what point during the consultation did you decide to use the POCT CRP device?
  - Did you use the POCT CRP device during the consultation, or did you ask patients to come back for sampling?
  - Did you feel that the use of the POCT CRP disrupted your personal workflow? If yes, in what way?
- Feasibility
  - Did you encounter any issues while using the POCT CRP device that prevented you from using it? If yes, which ones?
  - Did you notice any aspects that make it impossible to implement the POCT CRP device in your daily practice? If yes, which ones?
  - Were you able to use the POCT CRP device during every consultation where it was desired? If not, what were the reasons?
- Are there any topics that were not discussed during this focus group session that you feel should have been addressed?
- Of all the characteristics of the clinical decision support we discussed, which one is the most important to you? (Question for each participant)

# IV. Participant checking: results

Participant agreement with the themes was defined in three independent ways: (1) a median score ≥5; (2) an agreement proportion (score of at least 4) ≥75%; (3) a coefficient of variation (i.e., standard deviation divided by mean) ≤0.5. Consensus between participants was defined as an interquartile range (IQR) ≤1.75.

|  |  | Theme 1 | Theme 2 | Theme 3 | Theme 4 |
| --- | --- | --- | --- | --- | --- |
| Agreement | Median (at least 5) | 6 | 6 | 6 | 5 |
|  | Agreement proportion (at least 75%) | 100.00% | 100.00% | 100.00% | 95.24% |
|  | Coefficient of variation (at most 0.5) | 0.061 | 0.14 | 0.08 | 0.16 |
| Consensus | IQR ≤1.75 | 0.00 | 1.00 | 0.00 | 1.00 |

Reference: von der Gracht HA. Consensus measurement in Delphi studies: review and

implications for future quality assurance. Technol Forecast Soc Change

2012;79:1525–36.

# V.Full coding tree

**Theme 1: CRP POCT is an objective, non-invasive tool that reduces diagnostic uncertainty**

- Decision to perform CRP test
- Decision not to perform CRP test
- Tool to communicate with patient
- Diagnostic doubt / uncertainty management
- CRP test as an extra tool with clinical assessment
- Objectification
- Importance of clinical assessment or judgement
- Indications (for which the CRP test was used)
  - Usage in out of hours service
  - Usage in nursing home
  - Usage with children
  - Usage with home visits
  - Usage of the device outside of the study
  - Use of the device with people with acute cough
  - Follow up of the patient
  - Patients wanting repetitive CRP tests
  - Patient perception of the illness
- Comparison with non-electronical CRP test strips or general blood test

**Theme 2: Easy-to-use CRP POCT with reliable support integrates well in existing workflow**

- Installation/implementation of the CRP device
  - Device adaptation
- Device satisfaction
- Cooperation
  - Support by companies
  - Support by labs
  - Co-consultation
  - Technical problems
  - Time needed to resolve technical problems
- Workflow
  - Multitasking
  - Moment of performing CRP test
  - Person who performed CRP test
  - Location of the CRP device
  - Call with results
- Taking the blood sample
  - Amount of blood needed
  - Minimally invasive method
- Connection lab-EMR
  - Electronical medical record
  - Notation method of the result of the test in EMR
  - Link with health record in second line
- Quality control
- Young vs older physicians
- Frequency of use
- Duration of the entire testing process
- Disruption or prolongation of consultation

- Duration of the CRP test
- Storage time of the sample
- Speed of the CRP device
- Ease of use
- Multiparametric cassetes

**Theme 3: CRP POCT supports decisions and strengthens patient communication, fostering antibiotic stewardship**

- CRP value
  - Interpretation of the CRP value
  - Lack of evidence/guidelines
- Reliability of CRP result (+quality control)
- Confirmation of gut feeling or intuition
  - Reassurance
  - Surprising or unexpected results of CRP test
- False reassurance
- Avoid CRP dependence
- Starting or withholding antibiotics
- Tool to communicate with patients
- Added value for patients
- Added value for GPs
- More lab tests due to CRP test

**Theme 4: GPs expressed concerns regarding cost and reimbursement of CRP POCT4**

- Financial aspect - reimbursement

# VI. Dutch and French quotes

**Theme 1: CRP POCT is an objective, non-invasive tool that reduces diagnostic uncertainty**

| F2P4. “Et au niveau clinique, je pense que ça nous apporte quelque chose de très objectif. En médecine générale, c'est vrai qu'on est très fort sur le gut feeling et l'examen clinique, et de vraiment aussi ressentir un petit peu comment les gens se sentent, et ça permet une certaine objectivation des choses, en fonction de la CRP.” |
| --- |

| F4P1. “[...] hoe ik het persoonlijk vooral gebruikt heb, is denk ik een beetje tweeledig. Enerzijds vooral vanuit een optiek om om soms mensen te overtuigen om geen antibiotica te nemen, als er een vraag was naar antibiotica om een soort extra argument in de strijd te kunnen werpen en anderzijds soms bij randgevallen waarbij dat ik klinisch een beetje vreesde voor een ernstige infectie, om daar dan al dan niet uitsluiting of bevestiging van mijn klinisch aanvoelen, of van mijn klinische inschatting te hebben. |
| --- |

| F4P2. “Mensen vonden het leuk dat ze dan geen hele bloedafname moesten hebben. Mensen die schrik hadden van naalden, lieten da dan wel toe. Dan heb je iets, hè.” |
| --- |

| F1P8. “Ik denk: ja, nu hebben we ons gefocust op volwassenen met hoest. Ik denk, de meerwaarde bij kinderen is gigantisch, omdat je dan geen zware bloedafname moet gaan doen, die ze niet leuk vinden. Zo'n vingerprikje daar maken ze allemaal niks van.” |
| --- |

**Theme 2: Easy-to-use CRP POCT with reliable support integrates well in existing workflow**

| F3P8. “Ik merk ook dat in mijn praktijk vooral de jongere artsen het veel gebruikt hebben. Allez, ik heb het het meest gebruikt, denk ik (iedereen lacht). Ook wegens die diagnostische onzekerheid. De wat oudere artsen hebben het ietsje minder gebruikt, en zijn eerder op hun ervaring zijn afgegaan.” |
| --- |

| F2P12. “Nous on avait trois points de contact. On avait les points de contact des sociétés. J'ai dû faire appel pour des petits détails, pour rentrer dans les menus et sous menus et on avait une réponse immédiate. Et puis, il y en avait […] et […] pour le LUB, où je crois que j'ai quand même envoyé quelques petits messages au secours. Et puis, il y a […], qui assure la connectique et qui était très, très précieux. C'est clair que si ces machines sont là, il faut avoir ces gens précieux avec nous.” |
| --- |

| F2P9. “Alors que, en médecine générale c'est être à chaque fois conscient que les résultats qu'on a sont fiables ou pas, parce qu'on a mis en place ce calibrage qu'on tendance à ne pas faire chaque fois. Je viens de recommander ici, parce que je n’ai plus vos appareils. Donc j'ai dû recalibrer, et je me dis chaque fois: ‘Demain, demain, demain, et puis un patient arrive et il faut encore faire celui-là, et puis, mais on perd la fiabilité, parce qu'on n'a pas ce personnel que vous, que dans les autres laboratoires, cette rigueur que les laboratoires ont. Mais je pense que c'est une chose, une habitude à prendre. Après, je ne sais pas si c'est nécessaire. Peut-être ça, c'est le but du projet, un jour le rembourser. Je pense que ça peut être la popote interne, parce que, comme disait ma collègue très justement, c'est intéressant pour se rassurer en fin de journée, parfois quand on est en doute, pour se confirmer. Et je pense que même faut accepter parfois de perdre ce produit de réactif pour se rassurer et, bien soigner les gens.” |
| --- |

| F2P12. “En gros, au maximum, cinq minutes. Mais avec un temps qu'on peut en plus partager puisque la partie administrative on la fait pendant la partie administrative, la partie prélèvement on l'a fait pendant l'examen. La partie test on va chercher quelque chose, et on va rechercher le résultat après. Ce n'est pas cinq minutes qu'on doit rajouter à la consultation. C'est cinq minutes qu'il faut intégrer dans une consultation. Ça permet parfois de gagner du temps, quand il faut expliquer après pourquoi on n'a pas donné d'antibiotiques et là, on a une réponse immédiate. Parfois, ça nécessite des explications complémentaires. Bon ce n’est pas grave ça fait partie du jeu, hein.” |
| --- |

| F2P1. “Je pense que c'est quand même la facilité d’emploi. On n’est pas laborantin, donc c'est vrai que quand même, que ce soit facile. Bon, je reviens sur ce que j’ai dit tout à l’heure, je pense que la rapidité est importante, quand même, que ce soit quand même rapide et simple.” |
| --- |

**Theme 3: CRP POCT supports decisions and strengthens patient communication, fostering antibiotic stewardship**

| F1P8. “[…] het is inderdaad je verschiet soms in de twee kanten. Dat je denkt van: oké, dit zal wel meevallen maar we zullen toch een keer controleren, want het duurt bijvoorbeeld al iets langer, en dat je dan een torenhoog CRP hebt. Maar ook omgekeerd dat je iemand bij je hebt waarvan je zegt, die zit er nu echt wel doodziek uit en dat het allemaal zeer goed meevalt. En dan denk je: oké, dat is het verschil in de manier waarop dat mensen een ziekte beleven, he.” |
| --- |

| F1P1. “Ik kan mij alleen maar aansluiten bij de collega. Ja, ik zie direct ook heel veel meerwaarde. En het is wel waar, je moet blijven kijken naar je patiënt. En ook al is je testje oké, zeggen van, ‘Als het verslechterd kom dan terug’. Maar ik denk of dat je dat dan puur op basis van je kliniek [klinische onderzoek] doet of je doet dat ook met dat testje, ja. We mogen ons er niet volledig laten door leiden, maar het is inderdaad handig […] het geeft u meer geruststelling, dat je in de goede richting aan het denken bent.” |
| --- |

| F2P12. Ce que j'ai trouvé intéressant, c'est que ça a suscité beaucoup de co consultation. Ben c'est-à-dire que ma consœur faisait un test, elle avait un test qui était douteux, elle avait 27 et trouvait quand même que le patient n’était pas très bien. Donc, ‘Est-ce que tu peux venir écouter ? Me donner ton avis?’ Et ça, j'ai trouvé que c'était très intéressant. |
| --- |

| F1P8. “Zeker als we onze doelstellingen voor antibiotica een beetje willen halen en die voorschriften naar beneden krijgen. Ja, dat is toch een manier om dat op een gerustere manier te doen, waarbij zowel de patiënt als jij zelf toch meer gerust bent om te zeggen van, ‘Kijk, we gaan het niet doen.’ En ook omgekeerd. Ik denk dat het op termijn iets gaat worden dat bijna onmisbaar zal zijn voor bepaalde beslissingen. |
| --- |

| F1P1.b “Dus ik denk dat het zeker ook naar het opstarten van antibiotica een extra meerwaarde is, ook naar je patiënt toe. Als je op voorhand al heel duidelijk uitlegt, ‘Kijk, als het laag is, is het viraal, als het hoog, is het bacterieel’, en je komt dan terug en je zegt, ‘Kijk, het is laag’, dan hebben ze daar precies meer vertrouwen in.” |
| --- |

**Theme 4: GPs expressed concerns regarding cost and reimbursement of CRP POCT**

| F1P8. *“*Ik zou het een zekere meerwaarde vinden om dat, een toestel in in de praktijk te hebben. De vraag is natuurlijk: hoe ga je dat financieren? Oké kunt ge zeggen van ik ga aan elke patiënt de kostprijs van een testje doorrekenen. Dat kan maar dat is misschien toch niet optimaal, want als je het via het labo prikt, enkel CRP, dan is het in principe gratis. Dus als daar een oplossing voor gevonden wordt, denk ik dat de weg breed openligt om dat overal uit te rollen, natuurlijk. |
| --- |
| F3P2. “Ik vind zo de aankoop van het toestel, dat zou ik kunnen zien als een investering, dat je vindt van, ‘Ja, dat vind ik de moeite waard om te implementeren’. Maar ik vind niet per se dat dat moet doorgerekend worden aan de patiënt. Maar als elke cassette dan nog eens 5 euro is, ja, dan zit je wel al direct aan een serieuze investering. En dan denk ik van ja is dat eigenlijk voor ons om per se om te dragen? |

1. According to UZ Leuven policy, Study documents will be archived for at least 25 years following termination of Investigation. [↑](#footnote-ref-2)
